# Performance of secondary P-fertilizers in pot experiments analyzed by phosphorus X-ray absorption near-edge structure (XANES) spectroscopy

**DOI:** 10.1007/s13280-017-0973-z

**Published:** 2017-11-20

**Authors:** Christian Vogel, Camille Rivard, Verena Wilken, Andreas Muskolus, Christian Adam

**Affiliations:** 1 Division 4.4 Thermochemical Residues Treatment and Resource Recovery, Bundesanstalt für Materialforschung und –prüfung (BAM), Unter den Eichen 87, 12205 Berlin, Germany; 2 Synchrotron SOLEIL, St Aubin, 91192 Gif-Sur-Yvette, France; 3 Institute of Agricultural and Urban Ecological Projects affiliated to Berlin Humboldt University (IASP), Philippstraße 13, 10115 Berlin, Germany

**Keywords:** Phosphorus, Pot experiments, Secondary P-fertilizer, Sewage sludge ash, Soil, X-ray absorption near-edge structure (XANES) spectroscopy

## Abstract

A pot experiment was carried out with maize to determine the phosphorus (P) plant-availability of different secondary P-fertilizers derived from wastewater. We analyzed the respective soils by P K-edge X-ray absorption near-edge structure (XANES) spectroscopy to determine the P chemical forms that were present and determine the transformation processes. Macro- and micro-XANES spectroscopy were used to determine the chemical state of the overall soil P and identify P compounds in P-rich spots. Mainly organic P and/or P adsorbed on organic matter or other substrates were detected in unfertilized and fertilized soils. In addition, there were indications for the formation of ammonium phosphates in some fertilized soils. However, this effect was not seen in the maize yield of all P-fertilizers. The observed reactions between phosphate from secondary P-fertilizers and co-fertilized nitrogen compounds should be further investigated. Formation of highly plant-available compounds such as ammonium phosphates could make secondary P-fertilizers more competitive to commercial phosphate rock-based fertilizers with positive effects on resources conservation.

**Electronic supplementary material:**

The online version of this article (10.1007/s13280-017-0973-z) contains supplementary material, which is available to authorized users.

## Introduction

Phosphorus (P) is an essential element for all forms of life. It is necessary for metabolic processes (ADP/ATP) and is an integral part of the DNA molecule as well as the cell membrane. For those reasons, P in the form of phosphate is applied as an agricultural fertilizer to maximize plant growth (McLaughlin et al. 2011). Currently, organic (e.g., manure, compost, biogas digestates, and biosolids) and inorganic phosphate rock-based fertilizers are employed in agriculture. Due to the finite availability of phosphate rock resources (Gilbert 2009) and unstable phosphate rock prices (Mew 2016), sewage sludge (ash) has become an interesting secondary phosphate resource.

To improve the P uptake of plants during fertilization, knowledge about the availability of P to plants when fertilizer is added is necessary. Pot experiments are often performed to analyze the plant-availability of P-fertilizers to crops. Previous pot experiments with novel secondary P-fertilizers from wastewater treatment demonstrated very low P plant-availability of such fertilizers (Römer 2006; Cabeza et al. 2011; Nanzer et al. 2014; Severin et al. 2014; Vogel et al. 2015; Lekfeldt et al. 2016; Schnug and de Kok 2016). To further improve the plant-availability of secondary P-fertilizers, an understanding of the mechanisms of the reactions that these P-fertilizers undergo in soil is necessary. The work presented in this paper is meant to identify the P reaction products of secondary P-fertilizers applied to soil with nearly neutral pH before sowing and after harvest in agricultural pot experiments.

Phosphorus in soil has been previously analyzed by many researchers, who used various analytical methods such as sequential extraction, X-ray absorption near-edge structure (XANES), infrared, Raman, and ^31^P nuclear magnetic resonance spectroscopy (see reviews by Kizewski et al. 2011; Kruse et al. 2015). In this study, a combination of X-ray fluorescence (XRF) mapping and XANES spectroscopy was used to characterize the distribution and chemical status of P in soil. Determination of the P speciation in soil via XANES techniques has been discussed in several reviews (Hesterberg 2010; Doolette and Smernik 2011; Kizewski et al. 2011; Kruse et al. 2015). XANES spectroscopy has a significant advantage in that almost no sample preparation is necessary and the sample does not need to be modified by chemical treatment prior to analysis. However, if several compounds of an element are present in a sample, which is usually the case in soils, it can be difficult to determine the chemical state of the element even if linear combination fitting (LCF) has been applied. To distinguish between different P species, micro-X-ray fluorescence (µ-XRF) maps in combination with P K-edge micro-XANES (µ-XANES) spectra were collected from soils using an X-ray beam smaller than 1 µm^2^ (Lombi et al. 2006; Brandes et al. 2007; Giguet-Covex et al. 2013; Rivard et al. 2016). A combination of macro- and µ-XANES spectroscopy is suitable for determining both the chemical state of the overall soil P and of minor P phases, such as the applied fertilizer and its transformation products.

## Materials and methods

### Secondary P-fertilizers

The sewage sludge ash (SSA) that was used came from an incineration plant in Switzerland and originated from wastewater treatment plants that primarily used Fe-salts for phosphate precipitation. Thermochemical treatment of the SSA with magnesium chloride (hereafter referred to as SSA-Mg; Adam et al. 2009) and sodium carbonate (hereafter referred to SSA-Na; Stemann et al. 2015) was carried out in a rotary furnace (Thermal Technology, RT1700 and TL1100, Bayreuth, Germany) with a corundum and nickel-base alloy tube, respectively, for approximately 30 min at 950 °C under oxidizing (air, SSA-Mg) and reducing conditions (nitrogen gas, dry sewage sludge as reductive; SSA-Na). The major P phases in these P-fertilizers were magnesium phosphate (Mg_3_(PO_4_)_2_) and chlorapatite (Ca_5_(PO_4_)_3_Cl) in SSA-Mg (approximately 50:50%, Adam et al. 2009) and CaNaPO_4_ in SSA-Na (Stemann et al. 2015). Struvite (NH_4_MgPO_4_) came from a wastewater treatment plant in Germany. In the Airprex process (AirPrex 2016), sludge from an anaerobic digester is fed to an upflow reactor. The reactor is aerated, which leads to CO_2_ stripping, leading to a pH increase. After MgCl_2_ is dosed into the reactor, P precipitates in the mineral form of struvite. The precipitated struvite is separated at the bottom of the reactor. The chemical composition and the P-solubilities in water (P_W_), citric acid (P_CA_), and neutral ammonium citrate (P_NAC_) of these fertilizers are displayed in Table 1 and the characterization procedures described in the supplementary material. Triple Super Phosphate (TSP) was provided by the company Van Loon Hoeven (the Netherlands).

**Table 1:** Chemical composition and P-solubilities (P_NAC_, P_CIT_ and P_W_) of applied P-fertilizers

	Dry matter (%)	P_2_O_5_ (%)	P_NAC_ (%)	P_CIT_ (%)	P_W_ (%)	N (%)	K (%)	Mg (%)	Ca (%)	S (%)
Struvite	100	26.6	94	99	4	4.7	0.08	9.3	0.7	0.1
SSA-Mg	99.8	13.9	28	88	1	< 0.1	0.25	6.2	12.3	1
SSA-Na	100	12.9	99	100	10	< 0.1	0.58	1.4	9.9	1.1

### Pot experiment

A pot experiment using various secondary P-fertilizers and TSP as a reference was carried out with two soils (pH 4.9 and pH 7.1 in 0.1 M CaCl_2_) that had a low total P content. The soils were collected at two sites in Brandenburg, Germany, and included an acidic soil from Thyrow that had a sandy soil texture and a nearly neutral soil pH from Berge that had a loamy sand soil texture. These soils were mixed with equal amounts of quartz sand to lower the P content. The total P in the acidic soil was 114 mg kg^−1^, and the extractable calcium acetate lactate P (P_CAL_) was 38.0 mg kg^−1^. The total P in the nearly neutral soil was 117 mg kg^−1^ and the P_CAL_ was 17.0 mg kg^−1^. To achieve a nearly neutral pH value, lime was added to the latter soil (see Table S1 for additional data of both soils). The experiment was carried out in standard Mitscherlich pots with 6000 g of dry soil per pot. Four replicates per treatment (six for controls and TSP application) were established with three maize plants per pot of the Agro Max cultivar, Agromais, Germany. Phosphorus was added at 125 mg kg^−1^ soil (corresponding to 750 mg per pot and 188 kg ha^−1^) as powdered fertilizer (< 63 µm). The amounts of N, K, Mg, and S were adjusted individually for each P-fertilizer at the beginning of the experiment with nutrient solutions containing dissolved KNO_3_, NH_4_NO_3_, and MgSO_4_ to approximately 333 mg N, 333 mg K, and 33 mg S per kg of soil. Therefore, significantly less nitrogen was added to the pots of the N-containing struvite. Detailed information on the pot experiment were specified in the supplementary material.

Soil samples of approximately 10 g each were taken from the nearly neutral soil (i) directly after the soil was mixed with the P-fertilizer and nutrient solutions (before sowing) and (ii) after maize was harvested. The soil samples were subsequently air-dried for approximately 1 month for the spectroscopic studies. The calcium acetate lactate extractable P (P_CAL_) from the soils was extracted with calcium acetate lactate according to Schüller (1969). The total P was analyzed by ICP-OES (CEM Mars express, Kamp-Lintfort, Germany) after aqua regia digestion.

### Soil sample preparation for XANES analyses

For macro-XANES analysis, the soils were sieved into various particle size fractions (< 63 μm, 63–125 μm, 125–150 μm, 150–200 μm, > 200 μm, and additionally < 200 μm), in order to determine the size of the P-bearing minerals and/or the size of the particles on which phosphates are sorbed. Small amounts of these fractions were pressed into 3-mm-diameter pellets without a binding agent for P K-edge macro-XANES spectroscopy measurements. The particle fraction > 200 µm contained only quartz grains and was therefore not analyzed. For µ-XRF/µ-XANES analyses, soil fractions < 200 µm of each soil were dropped on a P-free adhesive tape (SSA-Mg before and after harvest) or embedded into a high-purity epoxy resin and the surface was subsequently polished with a 1-µm diamond polishing paste (unfertilized soil, SSA-Na before sowing and after harvest).

### References preparation for XANES analyses

The following reference compounds were used for XANES analyses: (NH_4_)_2_HPO_4_ (Merck, Darmstadt, Germany), NH_4_H_2_PO_4_ (J.T. Baker, Deventer, the Netherlands), Na_2_HPO_4_·2H_2_O (AppliChem GmbH, Darmstadt, Germany), Ca_5_(PO_4_)_3_OH, β-Ca_3_(PO_4_)_2_, CaHPO_4_·2H_2_O, Mg_3_(PO_4_)_2_·8H_2_O, (all Sigma-Aldrich, Steinheim, Germany), NH_4_MgPO_4_·2H_2_O, MgHPO_4_·2H_2_O, Ca(H_2_PO_4_)_2_·2H_2_O, phytic acid-Na salt, and adenosine triphosphate (ATP) (all Alfa Aesar, Karlsruhe, Germany). AlPO_4_ (cristobalite) was prepared from AlPO_4_ (Sigma-Aldrich, Steinheim, Germany) in a platinum crucible by thermal treatment (24 h) at 1300 °C in a muffle furnace (Nabertherm LH 15/14, Lilienthal, Germany). CaNaPO_4_ was thermally prepared from CaHPO_4_·2H_2_O and Na_2_CO_3_ in a platinum crucible at 1000°C (all chemicals: Sigma-Aldrich, Steinheim, Germany). Phytic acid-Ca salt, phytic acid-Fe salt, and phytic acid-Al salt were precipitated from dissolved phytic acid-Na salt (Sigma-Aldrich, Steinheim, Germany) with solutions of CaCl_2_, FeCl_3_, and AlCl_3_, respectively (He et al. 2006). All of these reference substances were finely ground and prepared as a homogeneous thin film on a P-free adhesive tape. Furthermore, phosphate adsorbed on ferrihydrite, dissolved organic matter (DOM), phosphate bound to DOM, and phosphate adsorbed on clay were prepared as described in Rivard et al. (2016).

### μ-XRF and XANES microspectroscopy

Micro-XRF maps and XANES spectra were collected on the ID21 X-ray microscopy beamline (Salomé et al. 2013) at the European Synchrotron Radiation Facility (ESRF, Grenoble, France). For detailed information of the setup see the supplementary material. The reference substances were prepared as thin films on adhesive tape. The XANES spectra were collected in transmission mode using a Si diode downstream of the sample. Two-dimensional μ-XRF elemental maps were collected at 2.25 keV (above the P K-edge) by raster scanning the sample with respect to the X-ray beam. Maps were collected using 250–500 ms data collection times per 1.0–5.0 μm step depending on the P concentration, value of the incident flux, and size of the P spot. To optimize the discrimination of the various XRF line contributions and, specifically, to remove the Si contribution to the P signal that originated from quartz and clay, the XRF spectra were batch fitted at each map pixel using the PyMCA software (Solé et al. 2007).

The P K-edge XANES spectra were collected from 2.13 to 2.20 keV with 0.2 eV energy steps in continuous scan mode. Depending on the P concentration and the matrix, 2–50 quick scan spectra of 0.1 s per energy step were recorded and averaged to optimize the signal-to-noise ratio (SNR). No radiation damage was observed during successive data collections. For each sample, 10–50 µ-XANES spectra were collected. The XANES spectra were reduced using the standard normalization procedure of the Athena package in the Demeter 0.9.20 system (Newville 2001; Ravel and Newville 2005). The *E*_0_ edge energy was chosen as the maximum of the first derivative of the data. The spectra were background corrected using a linear regression fit through the pre-edge region [*E*_0_ − 18 eV; *E*_0_ − 8 eV] and a polynomial regression fit through the post-edge region [*E*_0_ + 30 (± 2) eV; *E*_0_ + 47 eV], aiming at a horizontal post-edge signal and so that pre- and post-edge lines are parallel.

Linear combination fittings (LCF) were performed with Athena over the [*E*_0_ − 10 eV, *E*_0_ + 30 eV] range on the normalized bulk soil XANES spectra. LCFs were performed on the macro-XANES spectra of the fertilized soils. The choice of the standards used for the LCF was based on the nature of the applied fertilizer and on the nature of the μ-XANES spectra collected on the different samples. Furthermore, we performed correlation and scatter plots of the different elements for the µ-XRF data (see Fig. S4 to S12), but these plots did not evidence significant spatial correlation between P and other elements (Si, Al, Mg, and Na). Thus, the following spectra were retained: (NH_4_)_2_HPO_4_, NH_4_H_2_PO_4_, NH_4_MgPO_4_, Ca_3_(PO_4_)_2_, AlPO_4_, phytic acid-Na salt, ATP, DOM, phosphate sorbed to Al_2_O_3_, phosphate adsorbed onto clay, and phosphate adsorbed on ferrihydrite. The relative proportions of the components, whose number was limited to four, were forced to add up to 100%. From the remaining fits, the best fit was chosen, as seen by the lowest R. Some LCF calculation with different organic/adsorbed P did not differ significantly in the R-factor (due to only little spectral features of these compounds). Therefore, the specific type of organic/adsorbed P was difficult to identify.

## Results and discussion

### Pot experiment with maize on a nearly neutral and an acidic soil

**Fig. 1:**
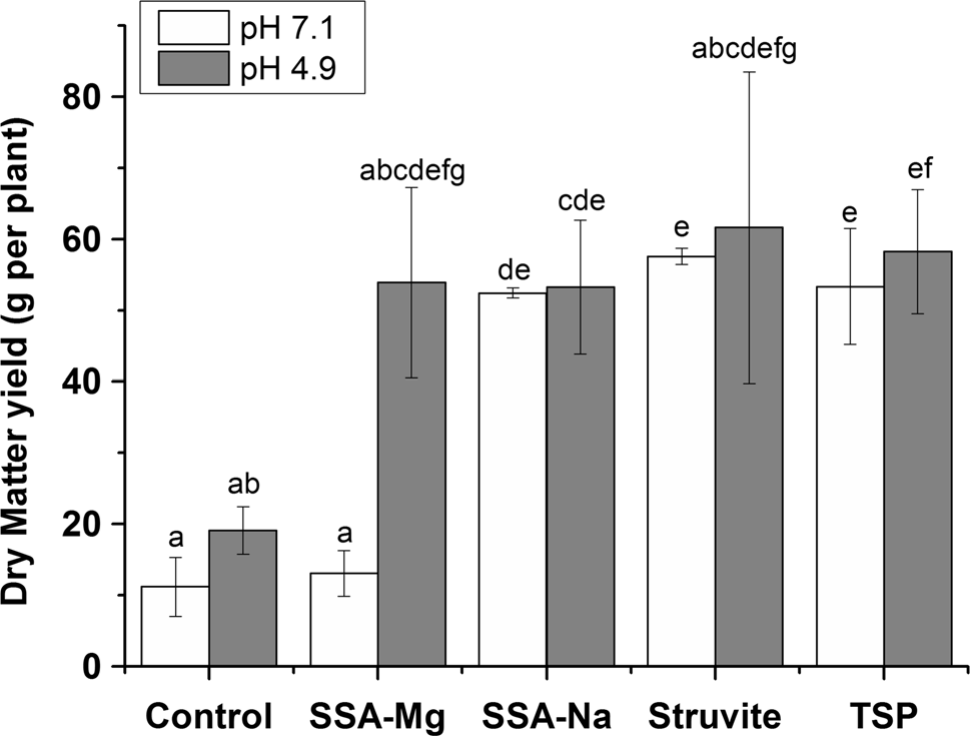
Dry matter yield of pot experiments with maize on the nearly neutral (7.1) and acidic (4.9) soils (as homogeneity of variances was only given for the nearly neutral soil, the Tukey test (*α* = 0.05) was used here; for the acidic soil, the Dunnett T3 test was used (*α* = 0.05))

The yield of maize grown on soil fertilized with various secondary P-fertilizers (SSA-Mg, SSA-Na, and struvite) was comparable to the yield of maize fertilized with TSP in acidic soil and significantly higher than the yield of the control (Fig. 1). By contrast, in nearly neutral soil, the yield of maize fertilized with SSA-Mg was much lower compared to that with the other fertilizers and was comparable to the control. Additionally, the P and N uptake of maize was in accordance with the yield and was much higher for SSA-Na, struvite, and TSP than for SSA-Mg in nearly neutral soil (Table 2), indicating that the P-fertilization performance of SSA-Mg was negligible. As for the yield, the P_CAL_ content (Table 3) of the soil fertilized with SSA-Mg was much lower than that with the other fertilizers, both before sowing and after harvest. To discover why the fertilizers performed differently, soil from the pot experiments with nearly neutral soil was analyzed in more detail by µ-XRF and P K-edge XANES spectroscopy.

**Table 2:** Phosphorus and nitrogen uptake during pot experiments with maize on the nearly neutral soil (pH 7.1)

	P uptake (mg P pot^−1^)	N uptake (mg N pot^−1^)
Control	47.6 ± 12.7	600.0 ± 177.2
SSA-Mg	57.1 ± 11.8	720.0 ± 141.5
SSA-Na	132.7 ± 5.1	1175.3 ± 93.0
Struvite	152.0 ± 13.4	1324.4 ± 145.9
TSP	135.4 ± 24.2	1273.6 ± 268.1

**Table 3:** Calcium acetate lactate extractable P (P_CAL_) of the nearly neutral soils (pH 7.1) from the pot experiments before sowing and after harvest

	Before sowing (mg kg^−1^)	After harvest (mg kg^−1^)
Control	17	12
SSA-Mg	43	32
SSA-Na	103	93
Struvite	122	78
TSP	109	79

### Macro-XANES spectroscopy of soil fractions < 200 µm

**Fig. 2:**
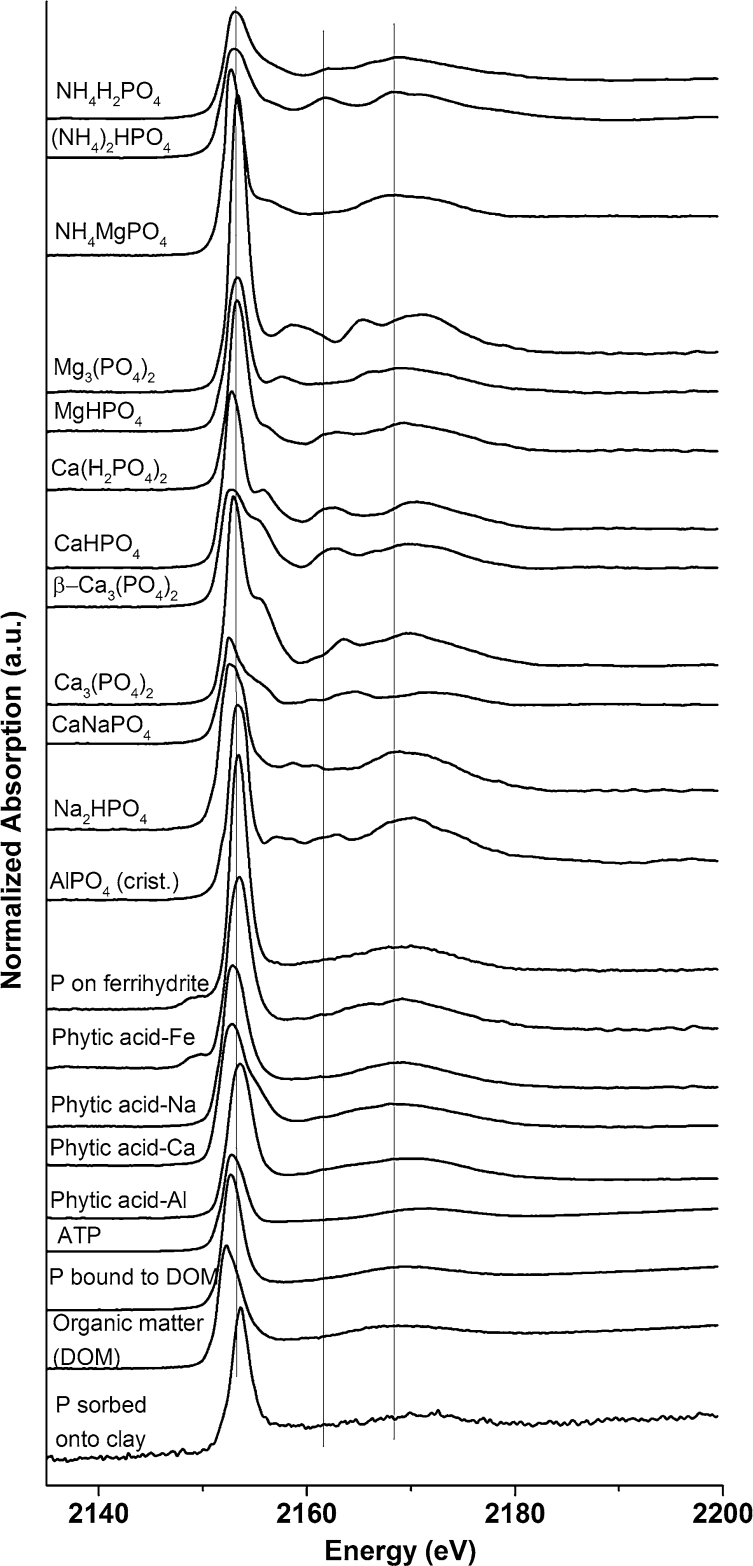
Phosphorus K-edge XANES spectra of various P reference compounds (crist. stands for cristobalite, ATP for adenosine triphosphate, and DOM for dissolved organic matter)

XANES spectra of various reference phosphate compounds were collected to interpret the soil measurements (Fig. 2). A detailed discussion on the different spectral features of these references is available in the supplementary material. The P K-edge macro-XANES spectra of the unfertilized soil and soil fertilized after harvest with SSA-Mg, SSA-Na, and struvite (all fraction < 200 µm) evidenced a difference in terms of SNR, which was attributed to the different amounts of P in the samples (Fig. S1). The XANES spectra of the unfertilized soil and the fertilized soils with struvite and SSA-Na after harvest did not show specific features, which suggested that the P in these three samples was probably mainly present as organic P and/or P bound to organic matter (OM) or adsorbed on other substrates, such as clay or non-crystalline aluminum (hydr)oxides (Khare et al. 2005). Only high amounts of P adsorbed to iron, manganese, or copper compounds in the oxidation state 3^+^ can be excluded because they show a little pre-peak (see Fig. 2 for iron compounds). However, due to possible superimposition of various P compounds in the XANES spectrum, a XANES spectrum can also occur which is close to organic P and/or P adsorbed on various substrates. Thus, LCF calculations and additional micro-XANES measurements were performed (see below). In contrast, the XANES spectrum of the soil fertilized with SSA-Mg differed slightly from the others, but the mass fractions of the P compounds were too low for a clear assignment; therefore, several different sieved soil fractions were analyzed.

### Different P forms in various soil fractions identified by macro-XANES spectroscopy

**Fig. 3:**
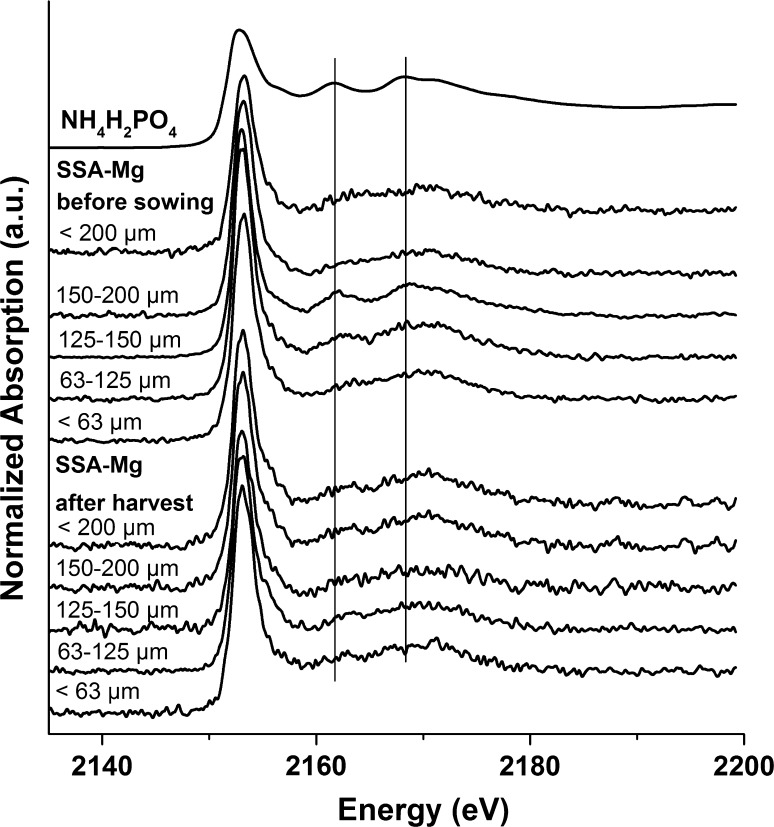
Phosphorus K-edge macro-XANES spectra of sieve fractions from the soils of the pot experiments with SSA-Mg before sowing (top) and after harvest (bottom)

The XANES spectra of soil fertilized with SSA-Mg before plant growth showed no specific features in the sieved fractions of 150–200 µm and < 63 µm (Fig. 3), suggesting that most of the P was organic P or P bound to OM, clay, or other substrates. By contrast, the sieved fractions of 125–150 and 63–125 µm had XANES spectra with specific features: a low-pronounced post-edge shoulder near 2156 eV and two oscillations in the post-edge region (2163 and 2167 eV). These features could be attributed to the presence of ammonium phosphates (NH_4_H_2_PO_4,_ (NH_4_)_2_HPO_4_) (see Fig. 2 for comparison). After harvest, the SNRs of the XANES spectra of the SSA-Mg fractions (Fig. 3, bottom) were lower than those from directly after fertilizer application, which might be due to P consumption by plants during growth. Nevertheless, significant differences were detected in the shape of the XANES spectra between samples before and after crop for the 125–150 µm sieve fraction, with a strong decrease in the two oscillations at 2163 and 2167 eV, which were attributed to ammonium phosphate species. This observation indicates the consumption of plant-available ammonium phosphate species in soil fertilized with the N-free fertilizer SSA-Mg, which is of course not in agreement with the results of the pot experiments, as they showed a negligible performance of SSA-Mg for P-fertilization. We assumed that we observed the reaction products of magnesium phosphates and ammonium stemming from N-fertilization. However, better fertilization performance due to this reaction was not observed, probably because only very small amounts were transformed. The sieved fractions of soil fertilized with SSA-Na did not show fraction-dependent differences in the XANES spectra and are not shown.

### Detection of ammonium phosphate formation by micro-XRF/XANES spectroscopy

**Fig. 4:**
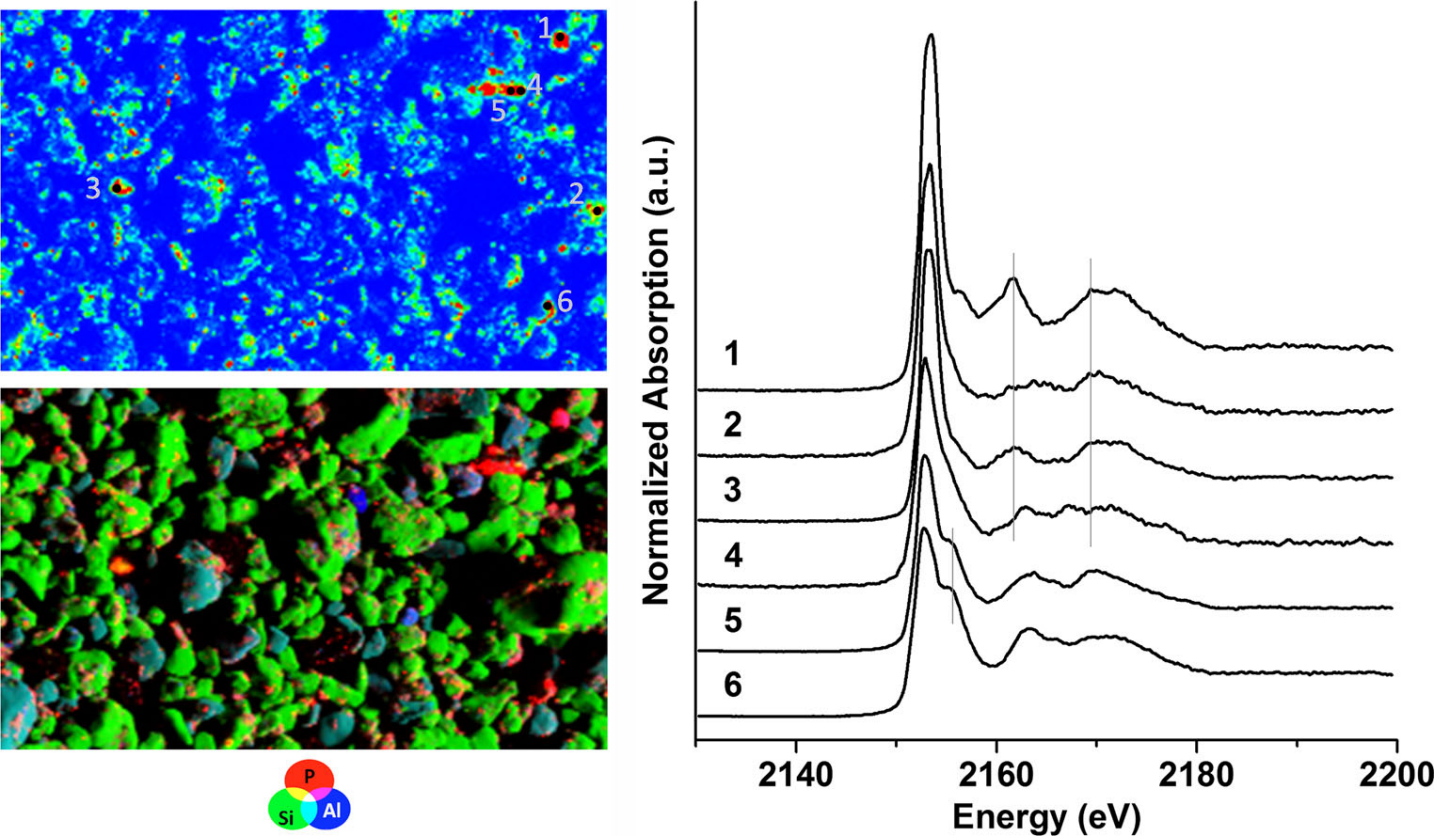
XRF map of P, Si, and Al (left bottom; 1 200 × 600 µm^2^, 5 µm step, color scale is arbitrary), P map with selected points of interest for µ-XANES (left top; red = high concentration, blue = low concentration), and µ-XANES spectra (right) of the fertilized soil with SSA-Mg after harvest (fraction < 200 µm) dropped on tape

In unfertilized soil, only organic P, P bound to OM, or other substrates, such as clay, P bound to Fe (adsorbed on Fe oxide/hydroxides or as Fe-phosphate), and P in apatitic minerals were found by µ-XANES spectroscopy (Fig. S2). As expected, the µ-XRF map of soil fertilized with SSA-Mg after harvest (Fig. 4, bottom left) showed that Si is a major soil compound that occurs in the form of SiO_2_ (quartz). µ-XANES spectra were collected from different P spots (Fig. 4, top left). Many spots showed XANES spectra close to ammonium phosphate (points 1–4). XANES spectra from other spots (5–6) had spectral features similar to those of apatitic species. The SSA-Mg P-fertilizer initially contained Mg-phosphate and chlorapatite. Mg-phosphates were not detected in soil by µ-XANES after fertilizer application (not shown) and after harvest. However, the presence of Mg-phosphate particles in the soil sample could not be formally excluded because the volume of the samples analyzed by µ-XRF/µ-XANES spectroscopy was relatively small. The P K-edge XANES spectra of chlorapatite, hydroxylapatite, and tricalcium phosphate are similar (Giguet-Covex et al. 2013); thus, a possible transformation from chlorapatite to tricalcium phosphate or hydroxyapaptite in soil were not detectable using only P K-edge XANES spectroscopy and XRF above the P K-edge techniques. µ-XRF investigations above the Cl K-edge could confirm this assumption. In this sense, Vogel et al. (2013) previously showed that chlorapatite can, under certain conditions, be transformed into hydroxylapatite in a slightly moist alkaline soil mixture which was analyzed by Raman microspectroscopy (pH 7.9).

**Fig. 5:**
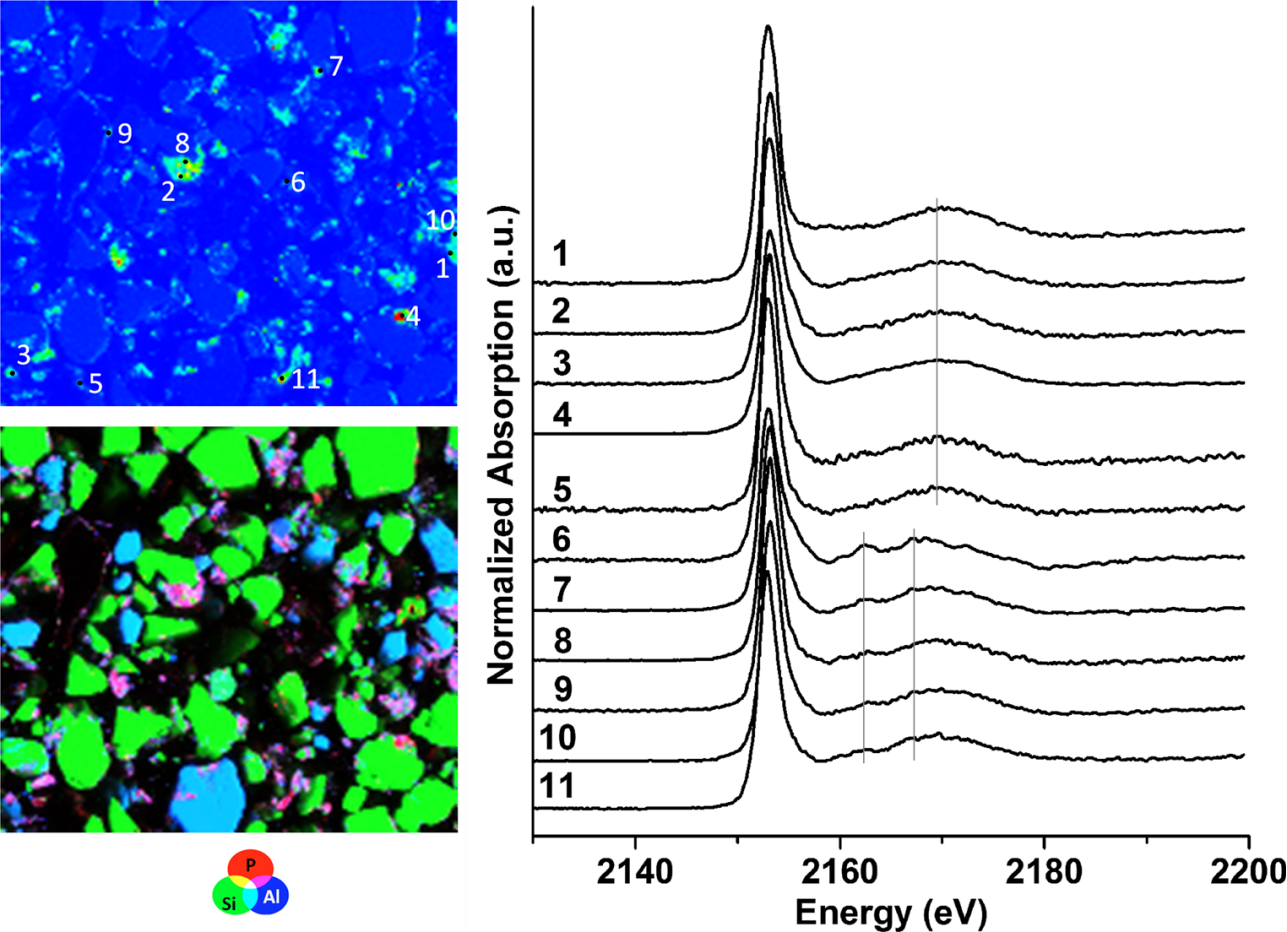
XRF map of P, Si, and Al (left bottom; 800 × 710 µm^2^, 5 µm step, color scale is arbitrary), P map with selected points of interest for µ-XANES (left top; red = high concentration, blue = low concentration), and µ-XANES spectra (right) of the fertilized soil with SSA-Na after harvest (fraction < 200 µm) embedded into resin

In soil fertilized with SSA-Na before sowing, besides organic and adsorbed phosphates, µ-XANES spectra with small pronounced post-edge shoulders at 2163 and 2167 eV attributable close to ammonium phosphate were collected (see Fig. S3). Many µ-XANES spectra of soil fertilized with SSA-Na after harvest (Fig. 5, points 7–11) showed spectral features that resembled NH_4_H_2_PO_4_. However, the pronounced post-edge shoulders were significantly weaker than for the SSA-Mg soil samples after harvest. Furthermore, spectra close to organic P, P bound to OM (points 4–6), or P adsorbed on clay minerals spectra (points 1–3) were also collected. In contrast to the soil fertilized with SSA-Mg, no apatitic P was found in soil fertilized with SSA-Na after harvest in the investigated volume. Because P bound in the apatitic form is not bioavailable in neutral soil, this observation could explain the better performance of P-fertilization of SSA-Na compared to SSA-Mg.

### Linear combination fitting of macro-XANES spectra

Because of the low P content, which was close to the detection limit of the beamline, no acceptable LCFs were obtained for the unfertilized soil XANES spectrum (not shown). By contrast, the macro-XANES spectra of fertilized soils had a higher SNR. Thus, based on the nature of the applied fertilizer and on the nature of the previous collected μ-XANES spectra, LCFs for the macro-XANES spectra of the fertilized soils were performed. Because the XANES spectra of organic P and adsorbed P (to organic matter, clay, or non-crystalline aluminum (hydr)oxides) showed only very slight differences, a change of the type of these organic/adsorbed P in the LCF calculation often resulted in a non-significant change in the R-factor. Therefore, the approx. amount of organic/adsorbed P in the soil could be determined, but other organic or absorbed P compounds are also possible.

**Fig. 6:**
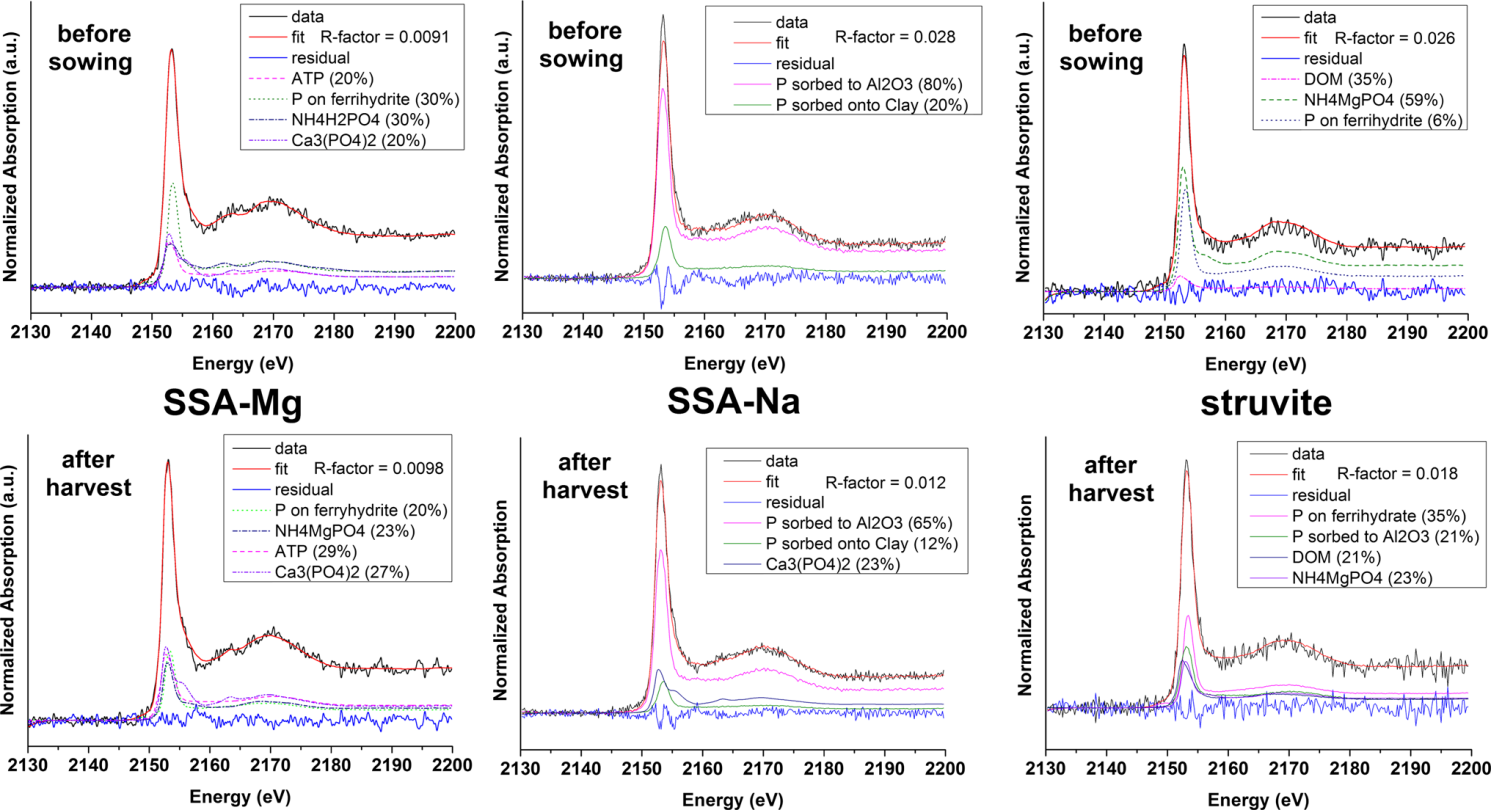
Phosphorus K-edge macro-XANES spectra of the three fertilized soils (with SSA-Mg, SSA-Na, and struvite) before sowing and after harvest and their corresponding best LCF

The best LCFs for soil fertilized with SSA-Mg before sowing (Fig. 6, top left) and after harvest (Fig. 6, bottom left) were obtained with the contribution of organic/absorbed P, ammonium phosphate, and insoluble Ca-phosphate. Because no ammonium phosphate was added to the pot experiments or in the fertilizer and was probably not in the unfertilized soil (due to the low P content, no acceptable LCFs could be obtained), this compound was presumably formed from the added N-free P source (SSA-Mg) and the nitrogen source (NH_4_NO_3_). Ammonium phosphates were also found as major P compounds in the LCFs of the more specific sieved soil fractions of SSA-Mg (see Figs. S13–S20). Not all of these LCFs could be perfectly fitted as the residual spectrum showed that a phosphorus compound was missing or subevaluated. The soil sample was taken immediately after the soil was mixed with nutrients, but it was air-dried for 1 month. Therefore, ammonium phosphate had time to develop in the soil sample. After harvest, the relative amount of insoluble Ca-phosphate increased (from 20 to 27%) and the amount of ammonium phosphate decreased (from 30 to 23%) with consumption during plant growth and possible phosphate fixation. We assumed that some of the Mg-phosphate reacted with ammonium compounds to form plant-available ammonium phosphate in the soil. The ammonium phosphates formed were partially consumed during plant growth, but that was not reflected in the yield and P uptake of the pot experiments. The P_CAL_ extractions (Table 2) showed that there was significantly more available P in the soil after harvest than in the control, which confirms that the fertilizer phosphate was only partially consumed.

By contrast, the best LCFs for the spectra of soil fertilized with SSA-Na before sowing and after harvest (Fig. 6, middle) do not include ammonium phosphates. The residual spectrum showed that the base of the white line on the high energy side was not correctly fitted, indicating that a phosphorus compound was missing or subevaluated. Furthermore, the amount of insoluble Ca-phosphate increased after harvest which is possibly due to phosphate fixation. The identification of ammonium phosphates in the µ-XANES spectra and their absence in the LCF indicate that this species is present and appears as hot spot in µ-XRF maps but occurs in a relatively low amount compared to other P species. The plant-available P is probably heterogeneously dispersed in soil and not present in large quantities in the volume that was studied.

The best LCFs of soil fertilized with struvite before sowing and after harvest (Fig. 6, right) were obtained via the contribution of organic/adsorbed P and NH_4_MgPO_4_. Similar to the observations for SSA-Mg, the amount of ammonium phosphate decreased from 59 to 23% with plant growth. The relatively high R-factor for this LCF mainly resulted from the low SNR of these spectra.

In summary, some of the phosphates contained in N-free SSA-Mg and SSA-Na possibly reacted with ammonium and formed plant-available ammonium phosphate in soil. The effect of ammonium phosphate formation was not reflected in the fertilization performance of SSA-Mg, which might be due to small amounts of transformed phosphates. The role of ammonium phosphate formation for SSA-Na in terms of its P-fertilization performance could not be clearly determined. Ammonium phosphate from struvite is directly plant-available (Bonvin et al. 2015). Chlorapatite present in SSA-Mg showed almost no reaction in nearly neutral soil. The poor plant-availability of apatite in nearly neutral soil could explain why the yield of maize for SSA-Na and struvite were much higher than for SSA-Mg in the pot experiments. This agrees with results of Nanzer et al. (2014), who also found poor plant-availability of apatitic P in neutral and alkaline soil.

### Effect of the chemical state of nitrogen on P uptake

Ammonium phosphates formed in soil appear to determine the plant-availability of P in the pot experiments. Thus, to adjust the elements in the pot experiments, not only the amount of P, N, K, S, and Mg but also their chemical state must be accurately analyzed. Table 4 shows the calculated NH_4_-N:NO_3_-N ratio of added N for the different P-fertilizers in the pot experiments. The K content was always adjusted with KNO_3_ first, followed by the N content with NH_4_NO_3_. Consequently, small differences in the NH_4_-N:NO_3_-N ratio of the control, SSA-Mg, SSA-Na, and TSP are evident. Because struvite already contains ammonium, which was charged in the calculation for the N adjustment, the struvite pots contained significantly more ammonium than the pots containing the other fertilizers.

**Table 4:** Calculated ratio (w/w%) NH_4_-N to NO_3_-N of applied nitrogen in pot experiments of various P-fertilizers

	NH_4_-N	NO_3_-N
Control	14.5	85.5
SSA-Mg	14.6	85.4
SSA-Na	14.8	85.2
Struvite	26.2	73.8
TSP	14.6	85.4

The yield of maize (Fig. 1) and the P and N uptake (Table 2) of struvite-fertilized plants were even slightly higher than for TSP-fertilized plants in the acidic and nearly neutral soils which might originate from a higher amount of ammonium in the pots. Römer (2006) also detected a higher yield for struvite compared to TSP in pot experiments. They assumed that the additional N from struvite was responsible for the increase because it was not considered in the N adjustment. However, this is not applicable in our study because the N content of struvite was considered in the calculation of the total added N. Furthermore, George et al. (2016) showed that small amounts of ammonium increase the P uptake of maize. In addition, Judel and Nitsche (1984), Apthorp et al. (1987), Thomson et al. (1993), and Bekele and Höfner (1993) found that ammonium fertilizer decreases the soil pH as a result of the nitrification of ammonium, which mobilizes soil phosphates. This could be another possible explanation for the results with struvite. The nitrification of ammonium in soil to nitrite and finally nitrate can be rapid (Bernal and Kirchmann 1992; Thomson et al. 1993) and would prevent the formation of ammonium phosphates. However, the formation of ammonium phosphates also seems to be a rapid reaction, as observed in the pot experiment over less than a month (air drying process).

The direct mixing of secondary P-fertilizers with N sources that contain a high amount of ammonium instead of nitrate could possibly provide a boost of the plant-availability of P. Rahmatullah et al. (2006) showed that fertilization with phosphate rock and ammonium compounds resulted in higher maize yields compared to phosphate rock application together with nitrate compounds. Furthermore, an even higher yield was obtained with the application of nitrification inhibitors.

## Conclusions

We used a combination of macro- and µ-XANES spectroscopy to determine the chemical state of the overall soil P and to identify locally concentrated P compounds in soil in pot experiments with secondary P-fertilizers. These measurements indicate ammonium phosphates in some soil samples. This is surprising, because the fertilizers SSA-Mg and SSA-Na are nitrogen-free. Thus, we suggest that some phosphates present in secondary P-fertilizers react after fertilizer application with co-fertilized ammonium compounds. Therefore, specific preparation of NP-fertilizers by granulation of secondary P-fertilizers with ammonium compounds and a nitrification inhibitor (Zerulla et al. 2001) could enhance the plant-availability of the fertilizer and make them more competitive with commercially available NP-fertilizers based on phosphate rock.

## Electronic supplementary material

Below is the link to the electronic supplementary material.

Supplementary material 1 (PDF 2635 kb)
