# Supplementary material for: Performance of secondary P-fertilizers in pot experiments analyzed by phosphorus X-ray absorption near-edge structure (XANES) spectroscopy
Source: Ambio. 2017 Nov 20;47(Suppl 1):62–72. doi: 10.1007/s13280-017-0973-z (PMC5722742; doi:10.1007/s13280-017-0973-z)

Ambio

Electronic Supplementary Material

This supplementary has not been peer reviewed.

Title: **Performance of secondary P-fertilizers in pot experiments analyzed by phosphorus X-ray absorption near edge structure (XANES) spectroscopy**

Authors: Christian Vogel, Camille Rivard, Verena Wilken, Andreas Muskolus, Christian Adam

Additional to Material and Methods:

Secondary P-fertilizer
The element mass fractions of the applied P-fertilizers were analyzed by ICP-OES (CEM Mars express, Kamp-Lintfort, Germany) after aqua regia digestion. The nitrogen content was determined after the combustion method (VDLUFA 3.5.2.7). Chemical extraction tests of these fertilizers were done with water (P_{W}; fertilizer/solution ratio 1:100, 30 min), 2% citric acid (P_{CIT}; fertilizer/solution ratio 1:100, 30 min) and neutral ammonium citrate (P_{NAC}; fertilizer/solution ratio 1:166, 60 min) according to the EU regulation No. 2003/2003. The P content of the solutions from the chemical extraction tests were analyzed by ICP-OES (CEM Mars express, Kamp-Lintfort, Germany).

Pot experiment
All of the Mitscherlich pots were placed in a randomized arrangement on tables sheltered from rain and birds by a transparent roof and a net. The average temperature during the day was 24°C and 13°C at night. The average sunshine duration was 8.5 h, and the air humidity was 69%. Daily watering kept the moisture content at 60% of the specific water holding capacity of the soil. The maize fresh matter from each pot was weighted 90 days after sowing, dried at 55°C and milled for sample analysis. The P mass fractions of the dried plant material were analyzed by ICP-OES (Thermo iCAP 6300 Duo, Dreieich, Germany) after microwave-assisted digestion (HNO_3/H_2O_2; CEM Mars express, Kamp-Lintfort, Germany).

µ-XRF and XANES microspectroscopy
The microscope was operated under a vacuum to minimize absorption and scattering by air. The X-ray beam was monochromatized using a fixed exit double-crystal Si(111) monochromator (0.4 eV resolution). The monochromator energy was calibrated against the first derivative maximum of tricalcium phosphate ($Ca_3(PO_4)_2$) at 2152.7 eV. Flux variations of the incoming beam were corrected by measuring the XRF from a Si_3N_4 substrate upstream of the sample with a photodiode. The beam size was reduced using a pinhole (200 µm diameter) for macro-XANES analysis or focused to 0.65×0.55 (h×v) μm^2 with a Kirkpatrick-Baez mirror for µ-XRF and µ-XANES analyses. The XRF signal was collected using an 80 mm^2 Bruker Si drift diode placed at a 49° angle with respect to the sample surface.

Additional to Figure 2:

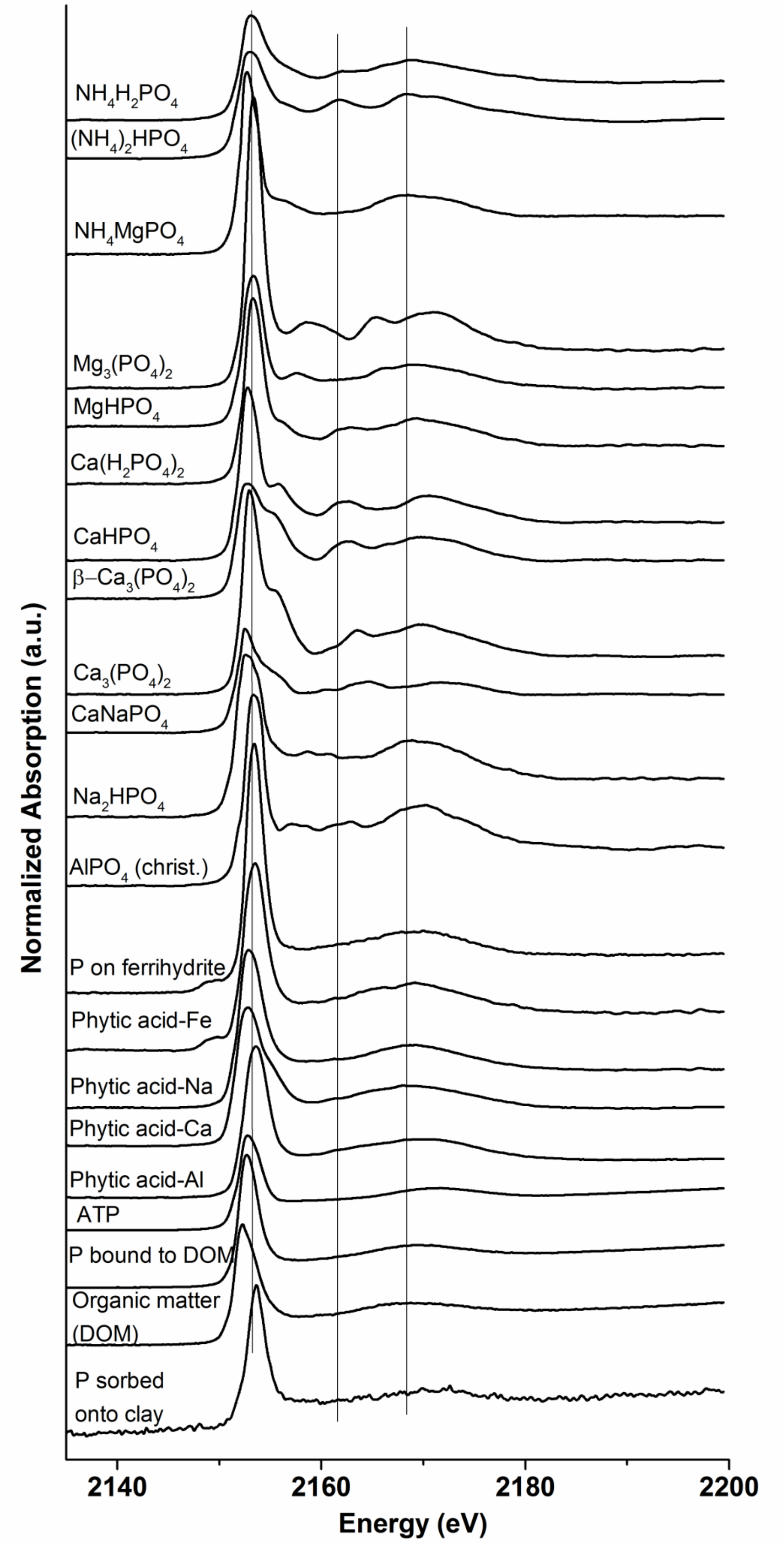

The ammonium phosphates $NH_4H_2PO_4$ and $(NH_4)_2HPO_4$ showed a pronounced post-edge shoulder at approximately 2157 eV as well as two characteristic oscillations at 2162 and 2167 eV. Mg-phosphates had a post-white line shoulder at ~2157 eV and one or two

oscillations between 2165 and 2180 eV. In contrast, the Ca-phosphates had a post-white line shoulder at ~2154 eV and two oscillations between 2160 and 2180 eV. This post-white line shoulder was present, but was less pronounced in the phytic acid-Ca salt spectrum, which indicated that this feature is characteristic for the Ca-phosphate group. The Na-phosphate showed a much broader white line. The white line of the Al and Fe-phosphates was slightly shifted towards a higher energy compared to the Ca- and Na-phosphates. Furthermore, the Fe-phosphates and phytic acid-Fe salt showed a pre-edge peak at ~ 2149 eV, characteristic of bound Fe^{3+}-phosphate. The XANES spectra of the adsorbed and organic P compounds showed a less specific fingerprint, with a white line and a broad oscillation, whose position and shape varied slightly as a function of the nature of the ligand (the white line for phytic acid-Fe, phytic acid-Al salts and phosphate adsorbed on clay were shifted towards a higher energy compared to phytic acid-Ca, phytic acid-Na salts, ATP, DOM and phosphate bound to DOM (see also Khare et al., 2005; Giguet-Covex et al., 2013; Kim et al., 2015; Prietzel et al. 2016).

References:

Giguet-Covex, C., J. Poulenard, E. Chalmin, F. Arnaud, C. Rivard, J.P. Jenny, J.M. Dorioz, 2013. XANES spectroscopy as a tool to trace phosphorus transformation during soil genesis and mountain ecosystem development from lake sediments. *Geochimica Cosmochimica Acta* 118:129–147.

Khare, N., D. Hesterberg, J.D. Martin, 2005. XANES investigation of phosphate sorption in single and binary systems of iron and aluminium oxide minerals. *Environmental Science and Technology* 39:2152-2160.

Kim, B., M. Gautier, C. Rivard, C. Sanglar, P. Michel, R. Gourdon, 2015. Effect of aging on phosphorus speciation in surface deposit of a vertical flow constructed wetland. *Environmental Science and Technology* 49:4903-4910.

Prietzel, J., G. Harrington W. Häusler, K. Heister, F. Werner, W. Klysubun, 2016. Reference spectra of important adsorbed organic and inorganic phosphate binding forms for soil P speciation using synchrotron-based K-edge XANES spectroscopy. *Journal of Synchrotron Radiation* 23:532-544.

Table S1: Chemical composition of both soils

	P mg kg^{-1}	C mg kg^{-1}	N mg kg^{-1}	K mg kg^{-1}	Mg mg kg^{-1}	Ca mg kg^{-1}
Soil pH 7.1	117	9450	430	410	6210	10920
Soil pH 4.9	114	1440	240	340	220	380

<u>Figure S1:</u> Phosphorus K-edge macro-XANES spectra of the unfertilized soil (bottom) and soils from the pot experiments fertilized with SSA-Mg, SSA-Na and struvite, respectively, analyzed after harvest (soil fraction <200 µm)

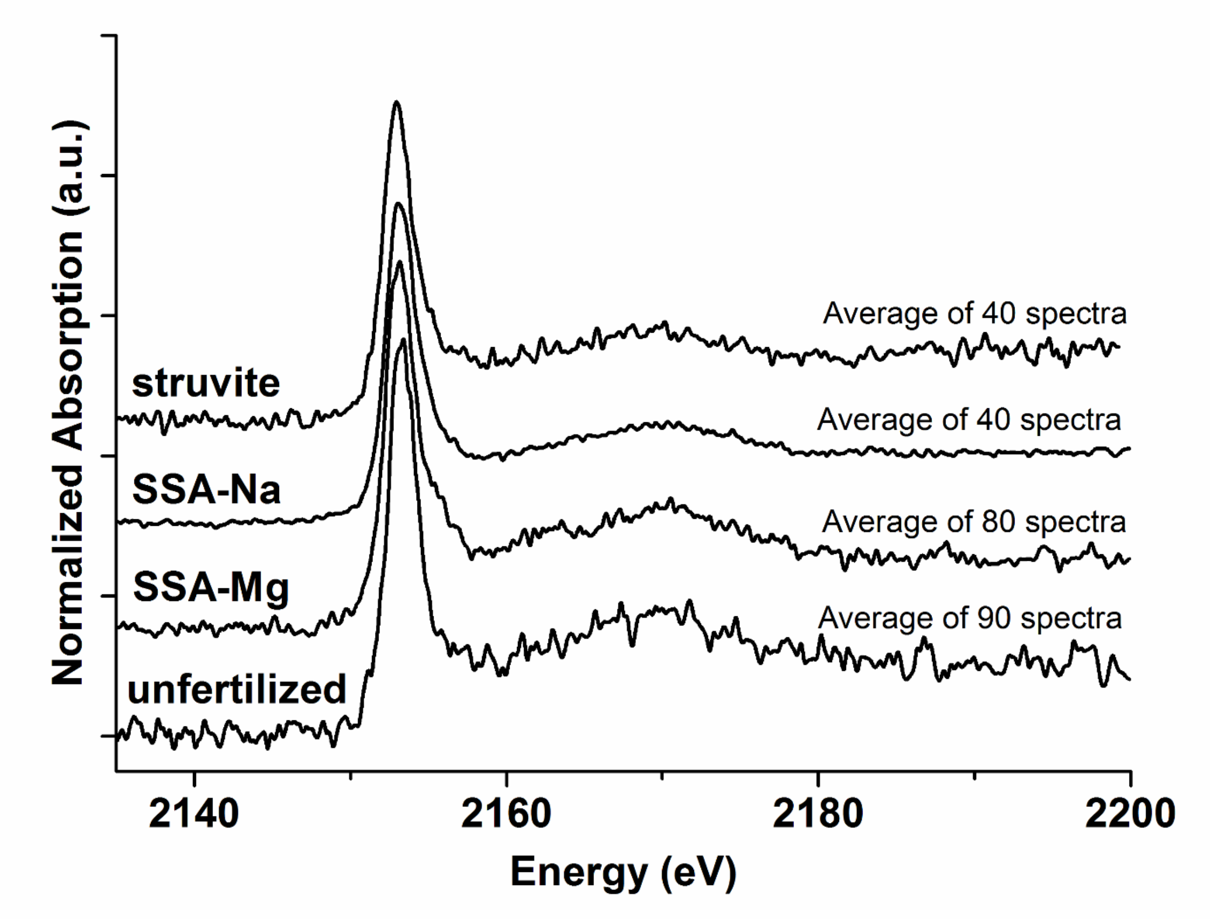

Figure S2: XRF map of P, Si and Al (left bottom; 1085 x 690 µm^2, 5 µm step, color scale is arbitrary), P map with selected points of interest for µ-XANES (left top; red = high concentration, blue = low concentration) and µ-XANES spectra (right) of the unfertilized soil (fraction < 200 µm) embedded into resin. Spectra 1-5 are attributed to organic or adsorbed phosphate and spectra 6 and 7 to apatite.

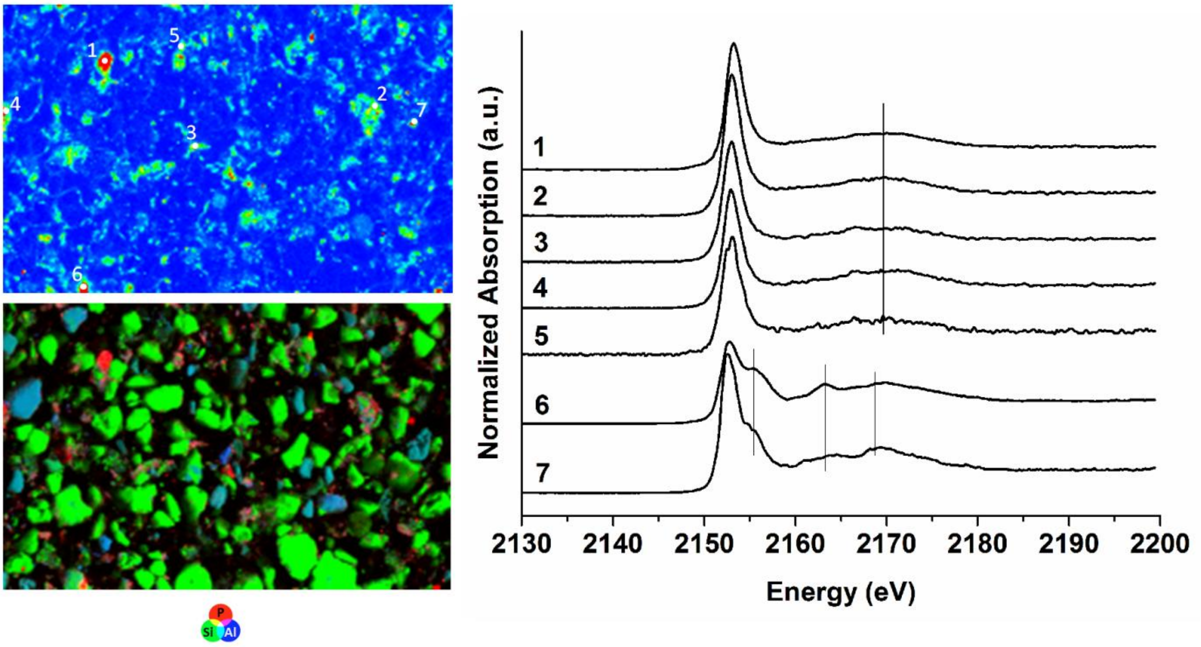

Figure S3: XRF map of P, Si and Al (left bottom; 1000 x 710 µm², 5 µm step, color scale is arbitrary), P map with selected points of interest for µ-XANES (left top; red = high concentration, blue = low concentration) and µ-XANES spectra (right) of soil from pot experiments of SSA-Na before sowing (fraction < 200 µm) embedded into resin. Spectra 1-5 are attributed to organic or adsorbed phosphate; spectra 6-9 to ammonium phosphate and spectrum 10 to apatite.

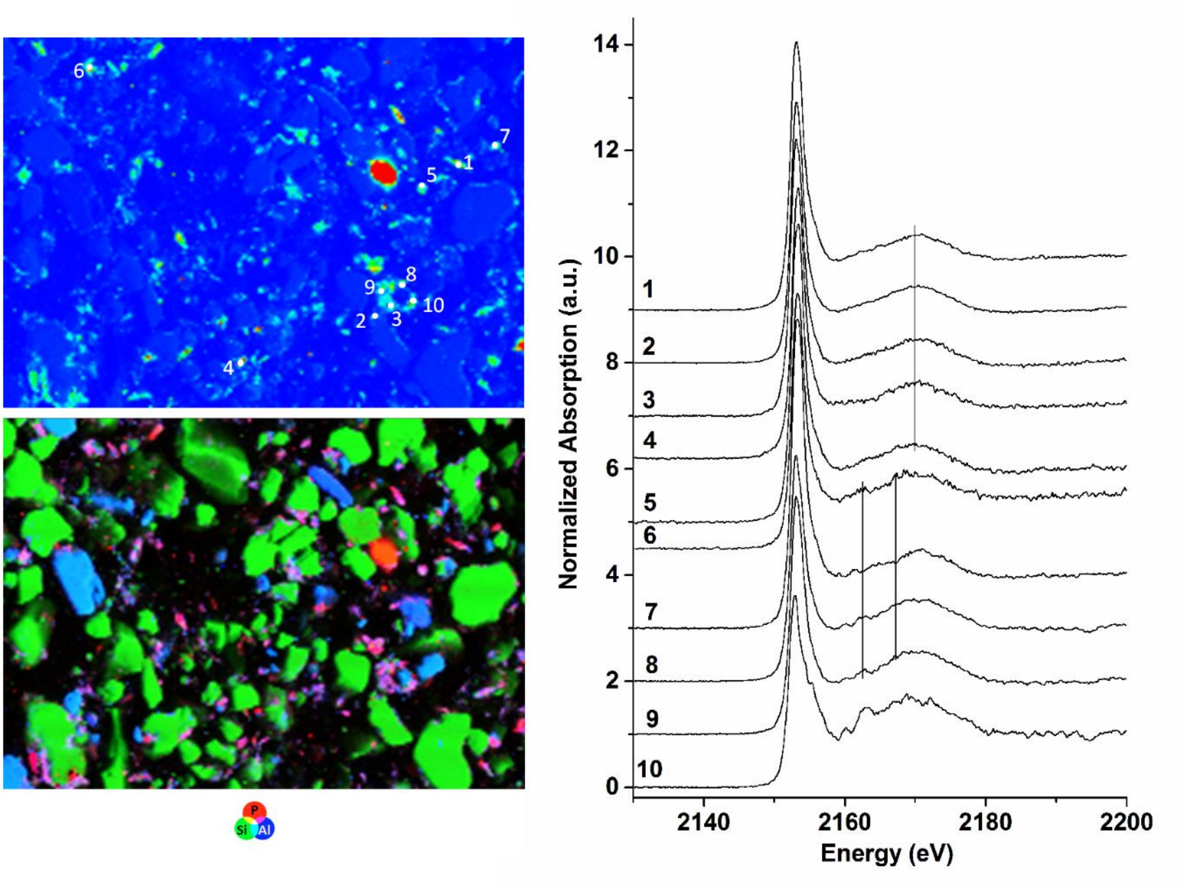

Figure S4: Elemental correlation maps for the unfertilized and fertilized soils (< 200 µm sieved fraction). Spatial resolution is 5 µm. The sample way of preparation (resin embedded or dropped on adhesive tape) is indicated for each sample. A common color scale is used for unfertilized and SSA-Mg samples (c1). A common color scale is used for SSA-Na samples (c2), with c2 = αc1 where α is a constant because the latter samples were analyzed during a second experiment run, without the same electron beam intensity in the synchrotron ring (and so without the same flux on the sample and same XRF detector position).

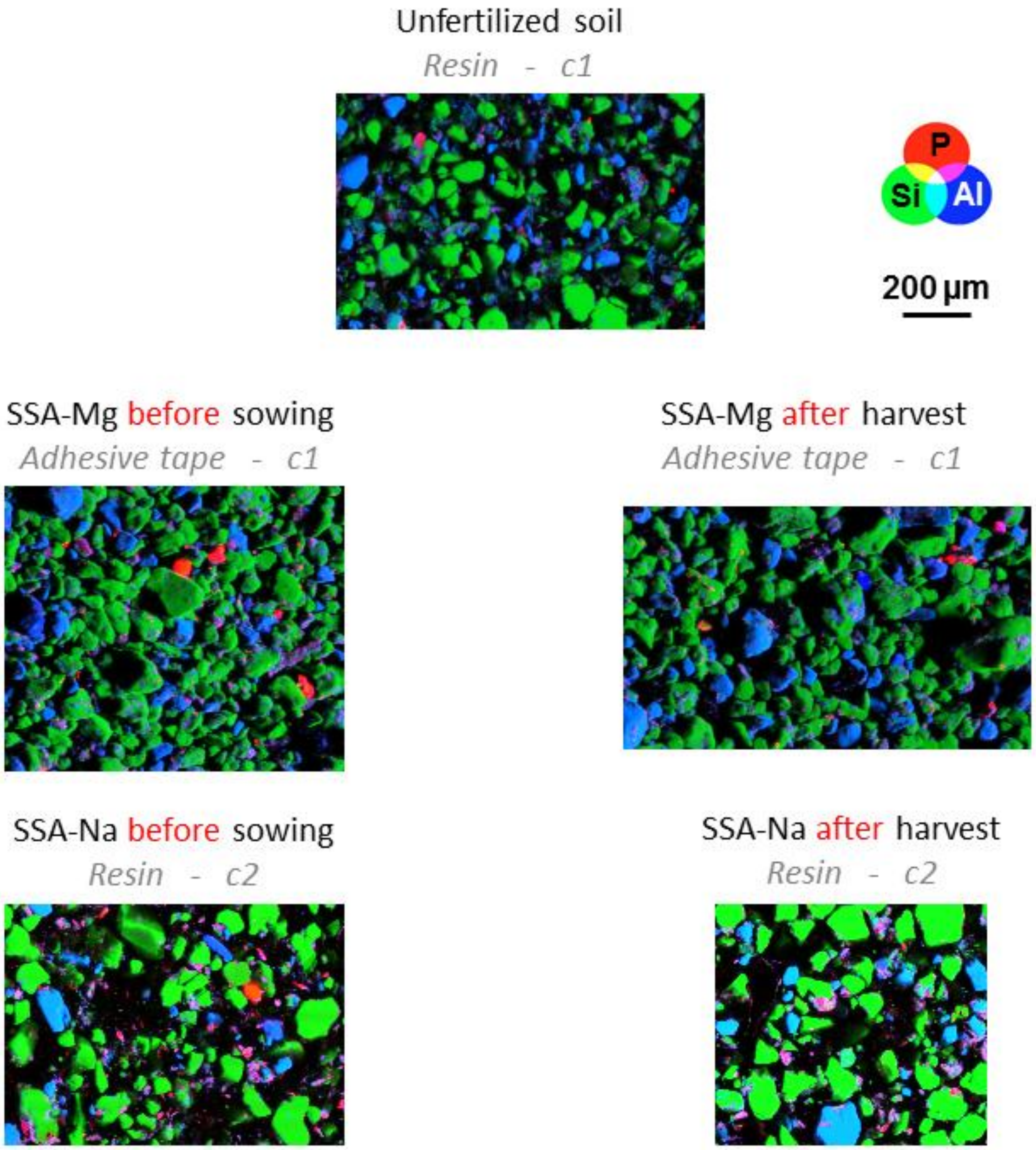

Figure S5: Elemental correlation maps for the SSA-Mg before sowing and after harvest (< 200 µm sieved fraction). Spatial resolution is 5 µm. The samples were dropped on adhesive tape. A common color scale is used for these two samples.

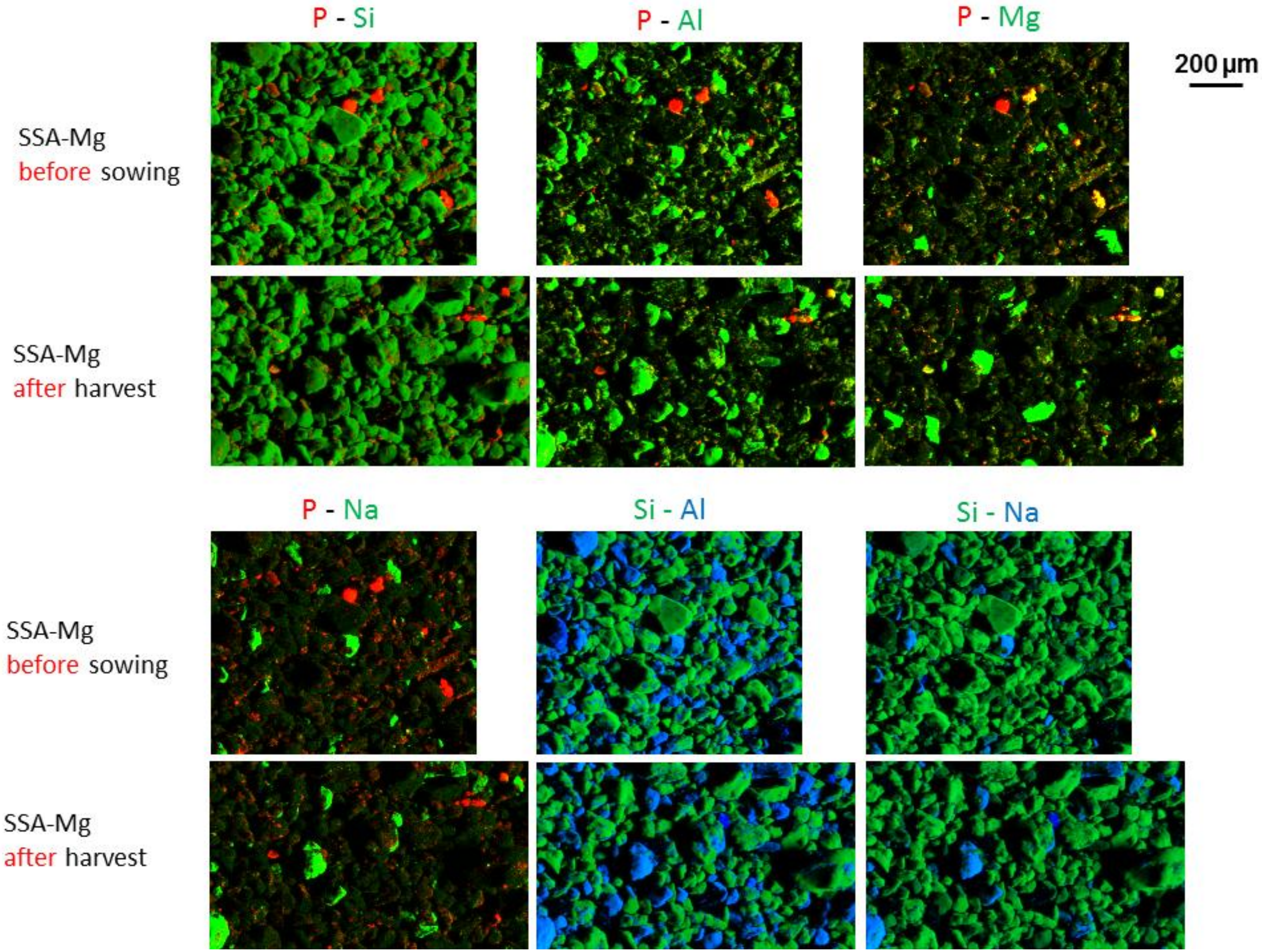

Figure S6: Elemental correlation maps for the SSA-Na before sowing and after harvest (< 200 µm sieved fraction). Spatial resolution is 5 µm. The samples were embedded in resin. A common color scale is used for these two samples.

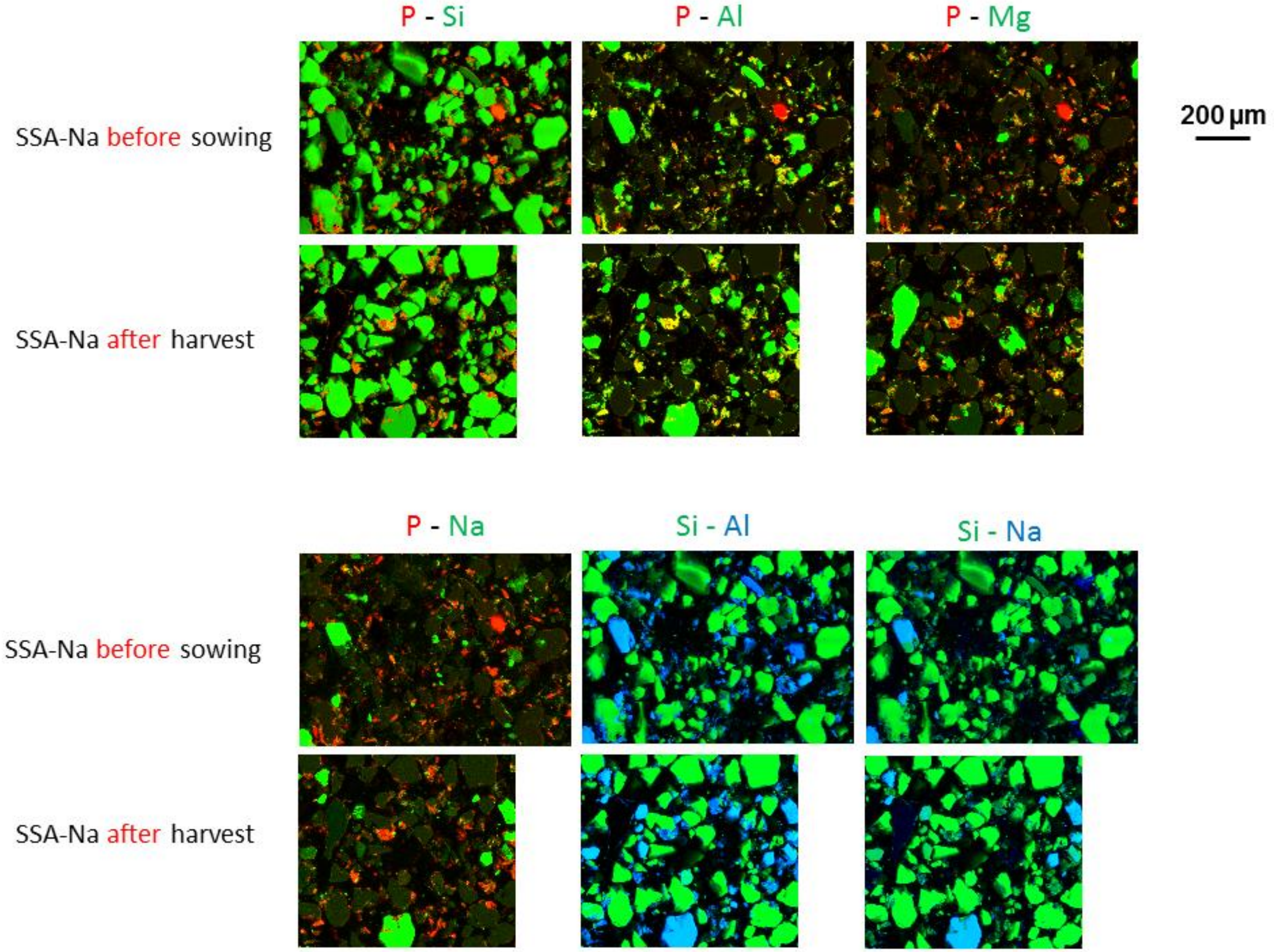

Figure S7: P-Si scatter plots of the µ-XRF data from the different soils. The points encircled in the red ellipse correspond to the P-rich hotspot indicated by the arrow on the map.

P - Si

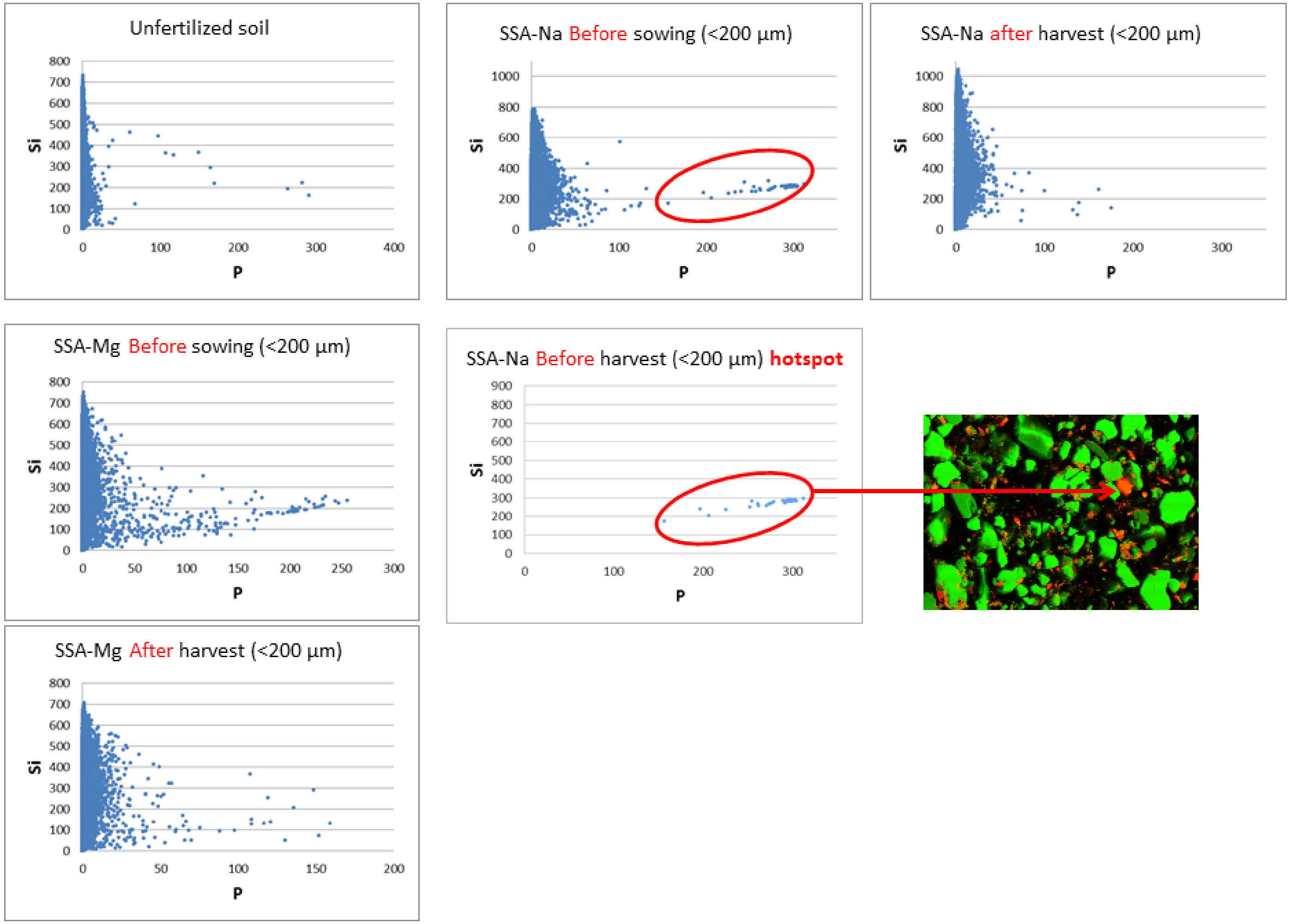

Figure S8: P-Al scatter plots of the µ-XRF data from the different soils

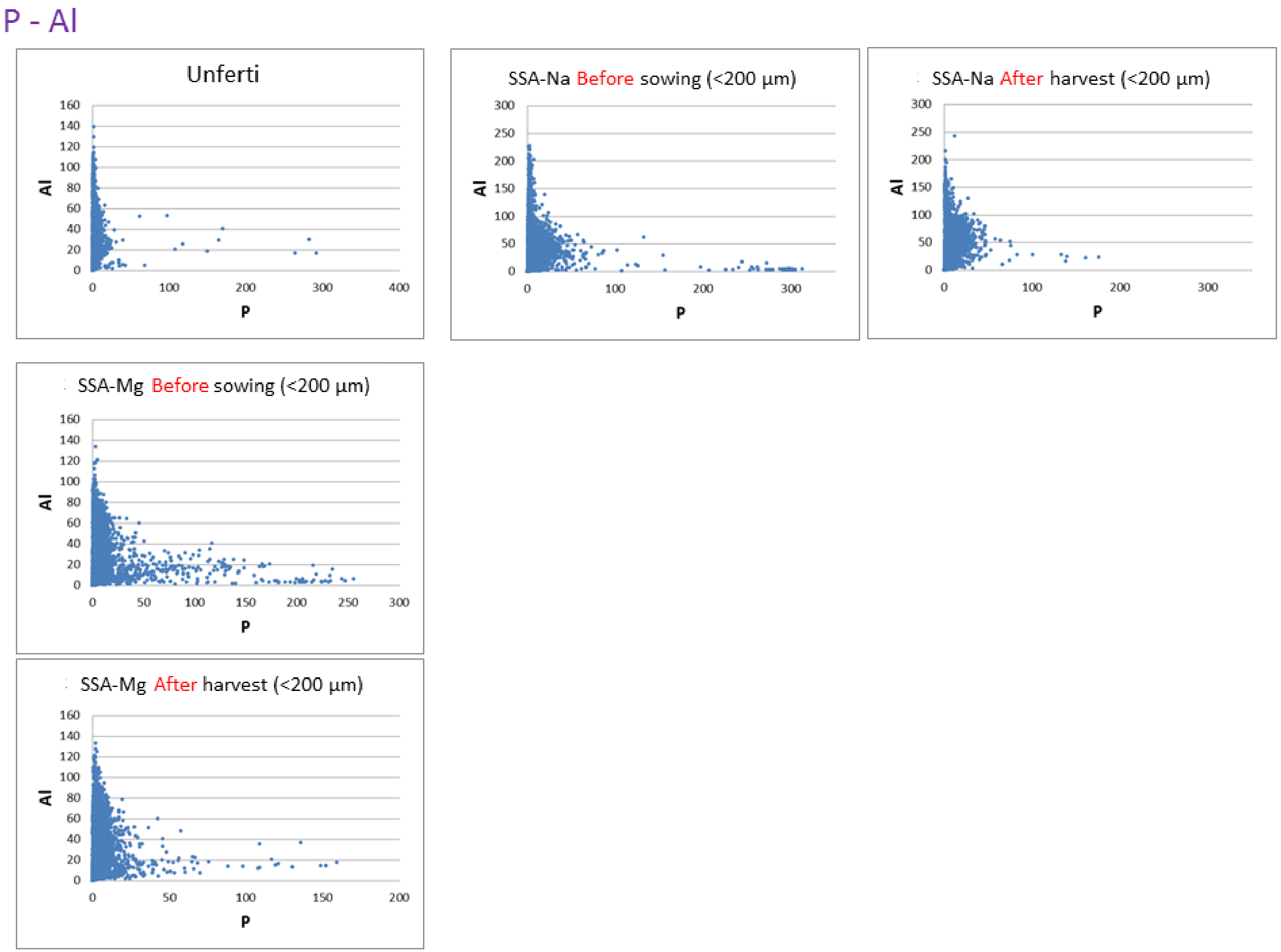

Figure S9: P-Mg scatter plots of the μ-XRF data from the different soils

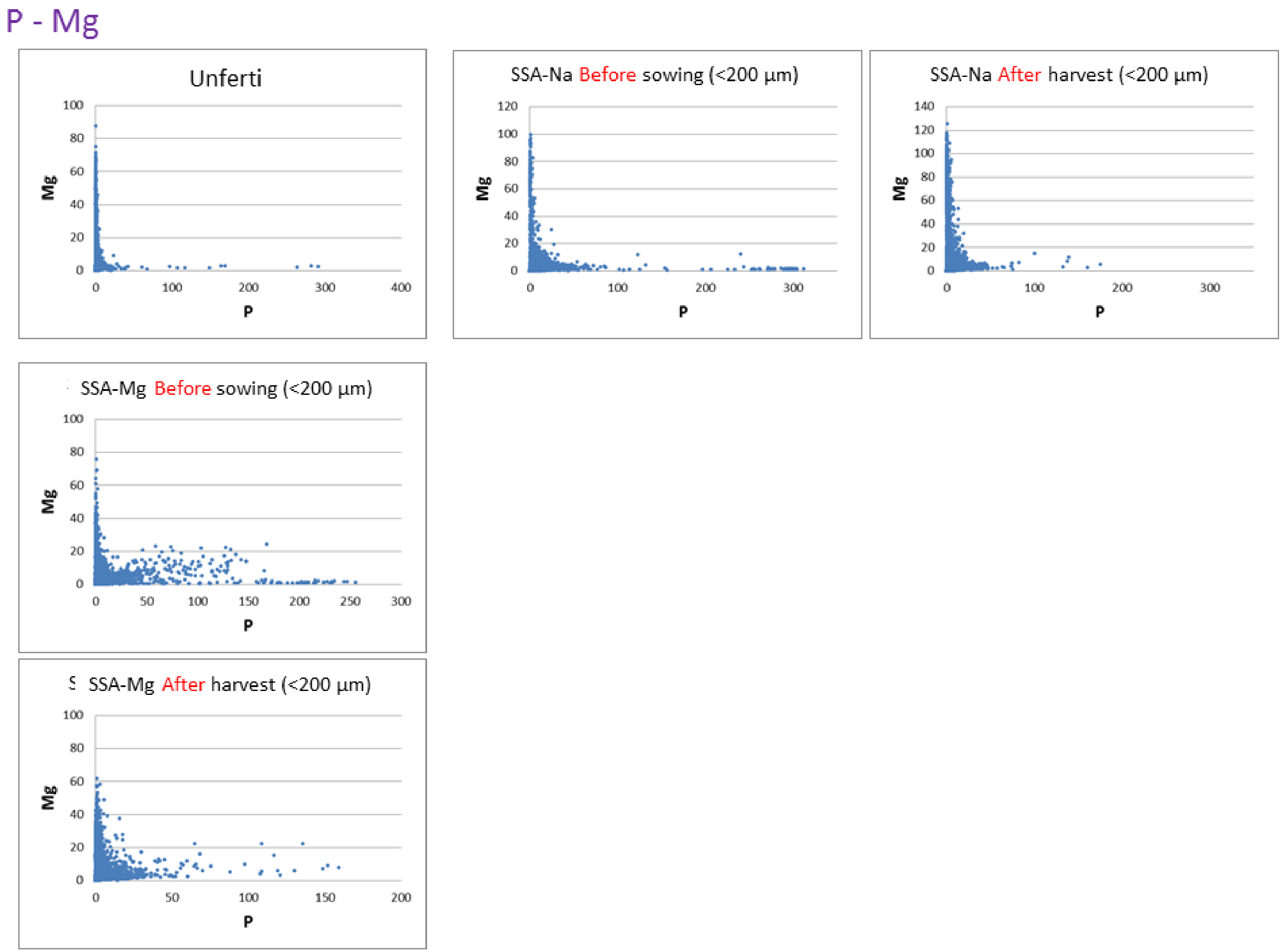

Figure S10: P-Na scatter plots of the µ-XRF data from the different soils

P - Na

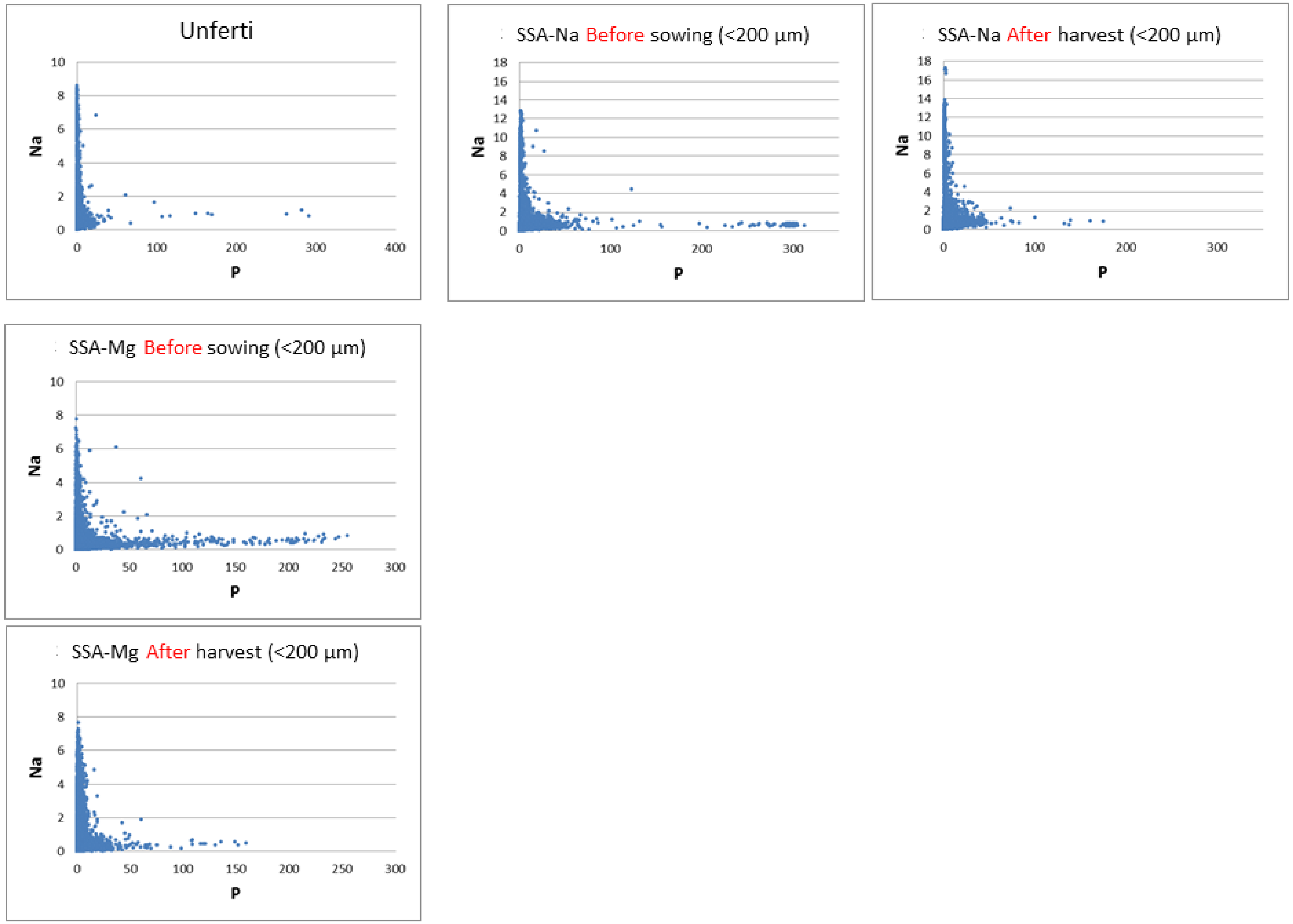

Figure S11: Al-Si scatter plots of the μ-XRF data from the different soils

Al – Si

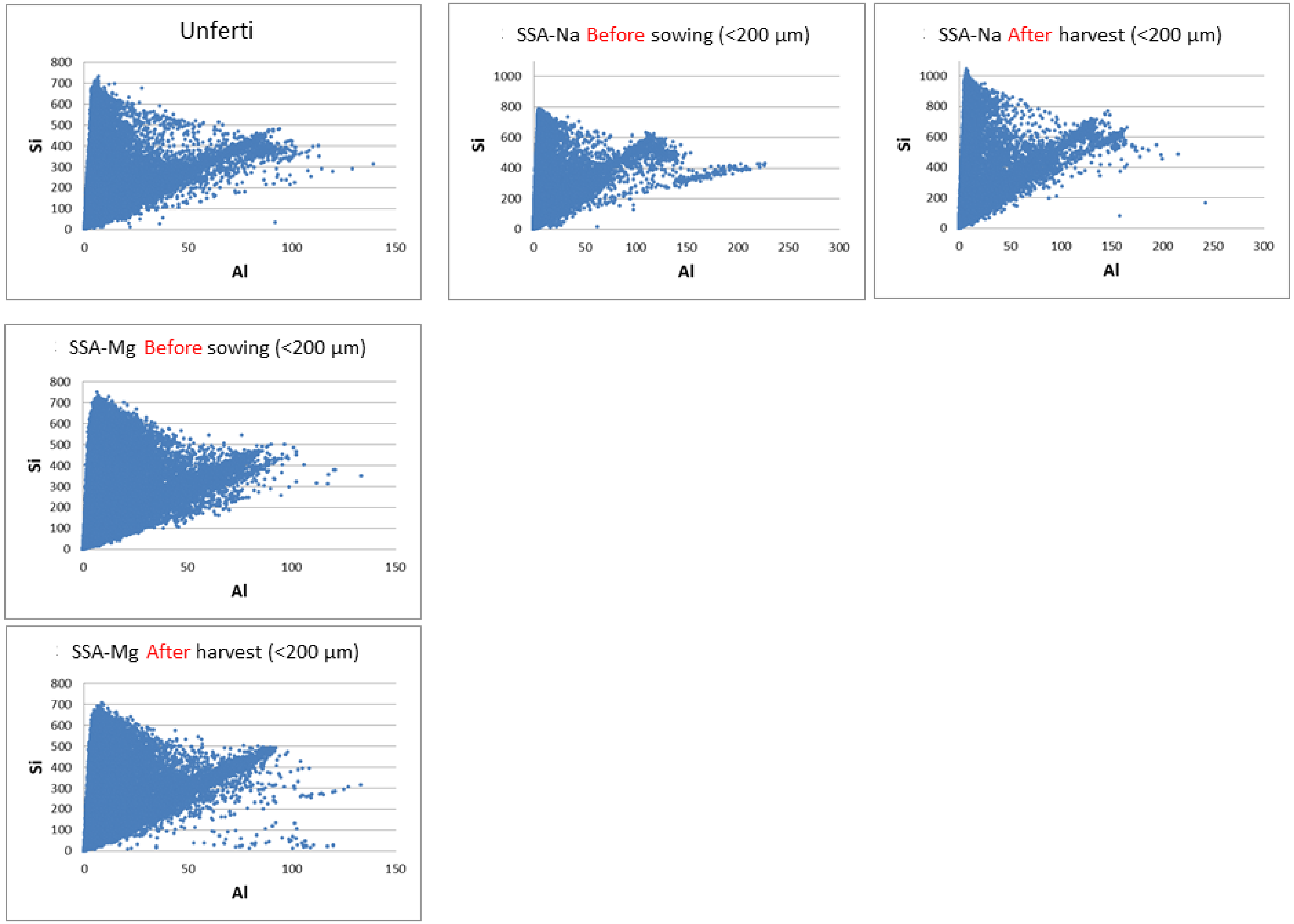

Figure S12: Na-Si scatter plots of the µ-XRF data from the different soils

Na – Si

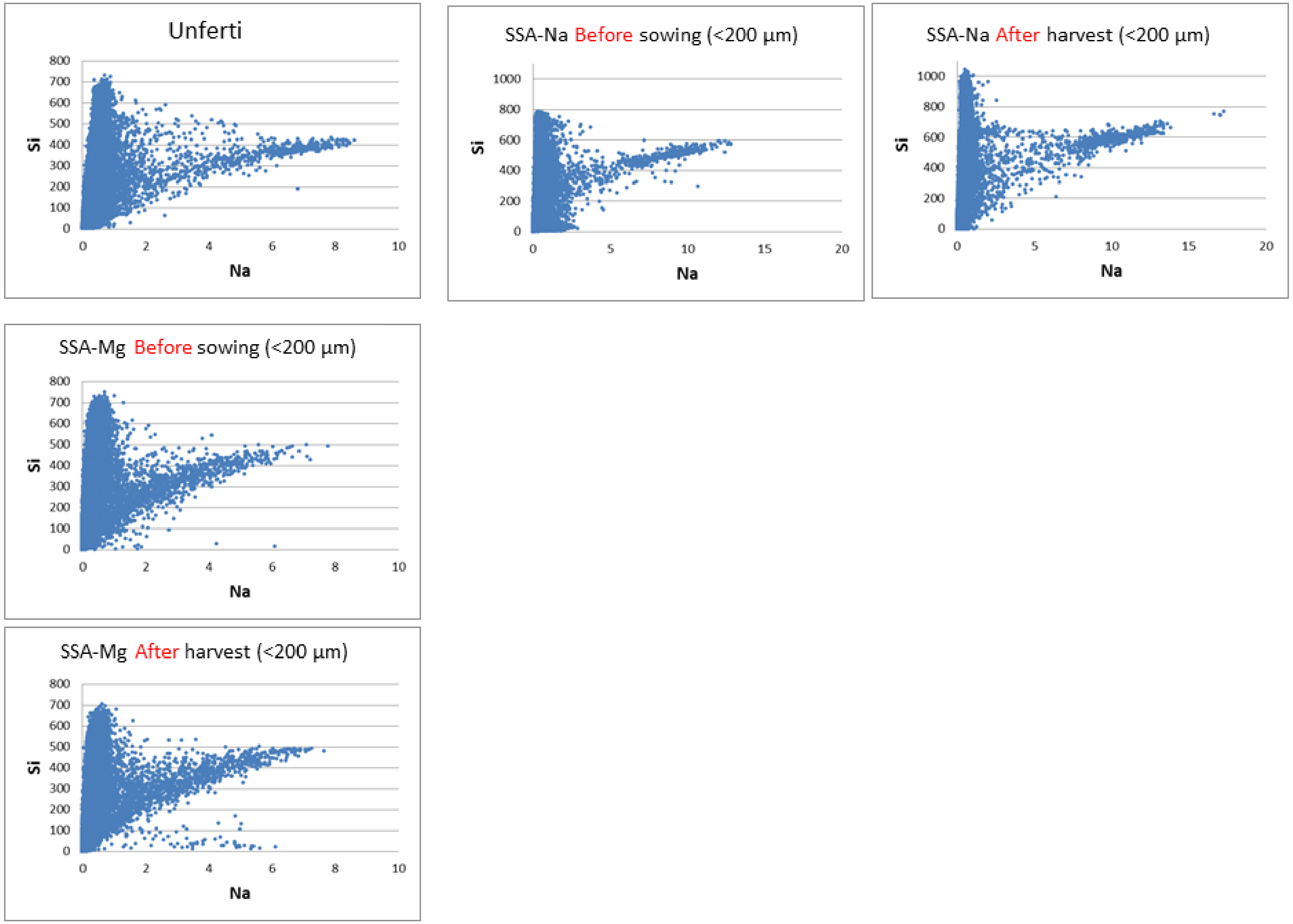

Best fits of LCFs of the sieved fractions of SSA-Mg before sowing and after harvest:

Figure S13:

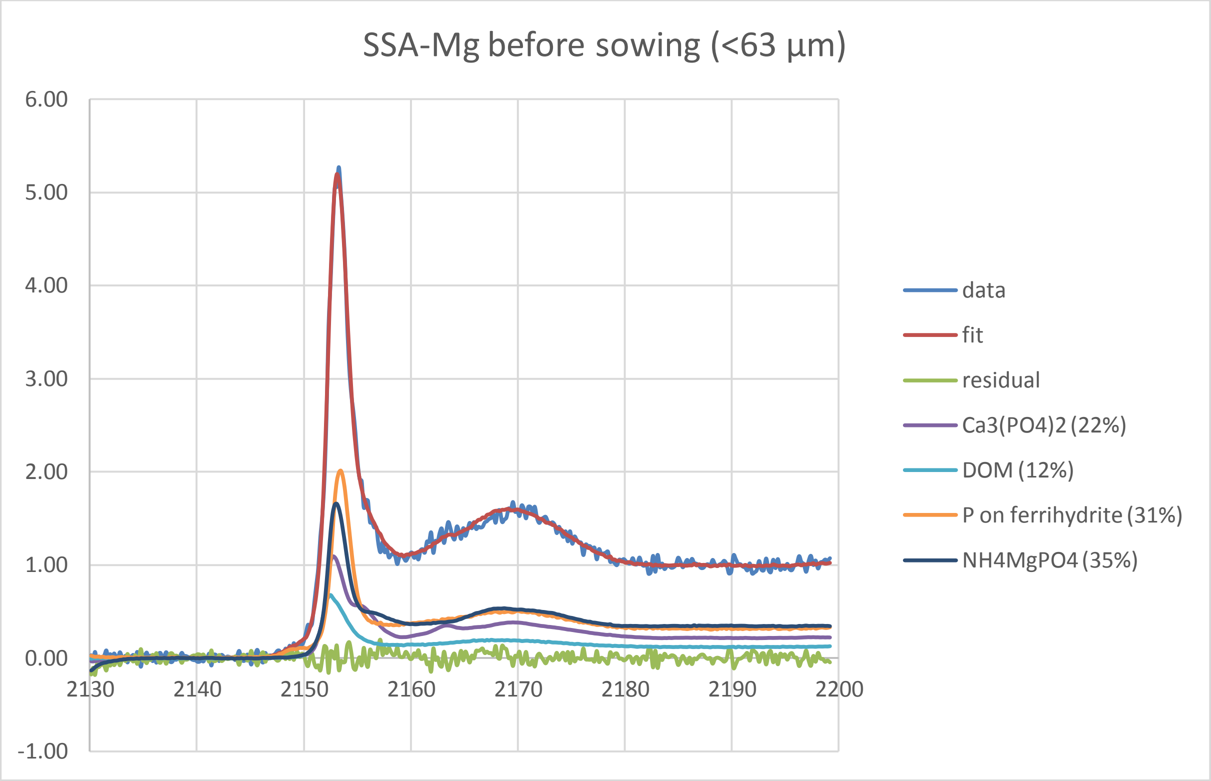

Figure S14:

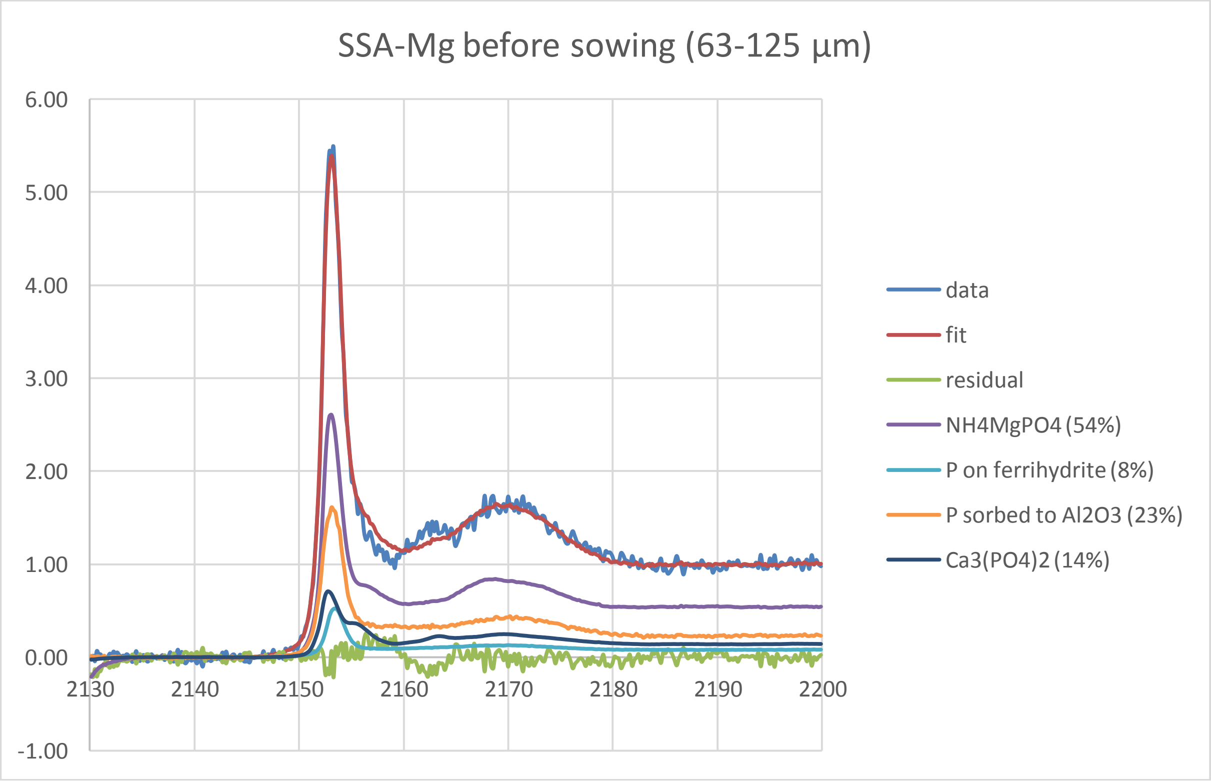

Figure S15:

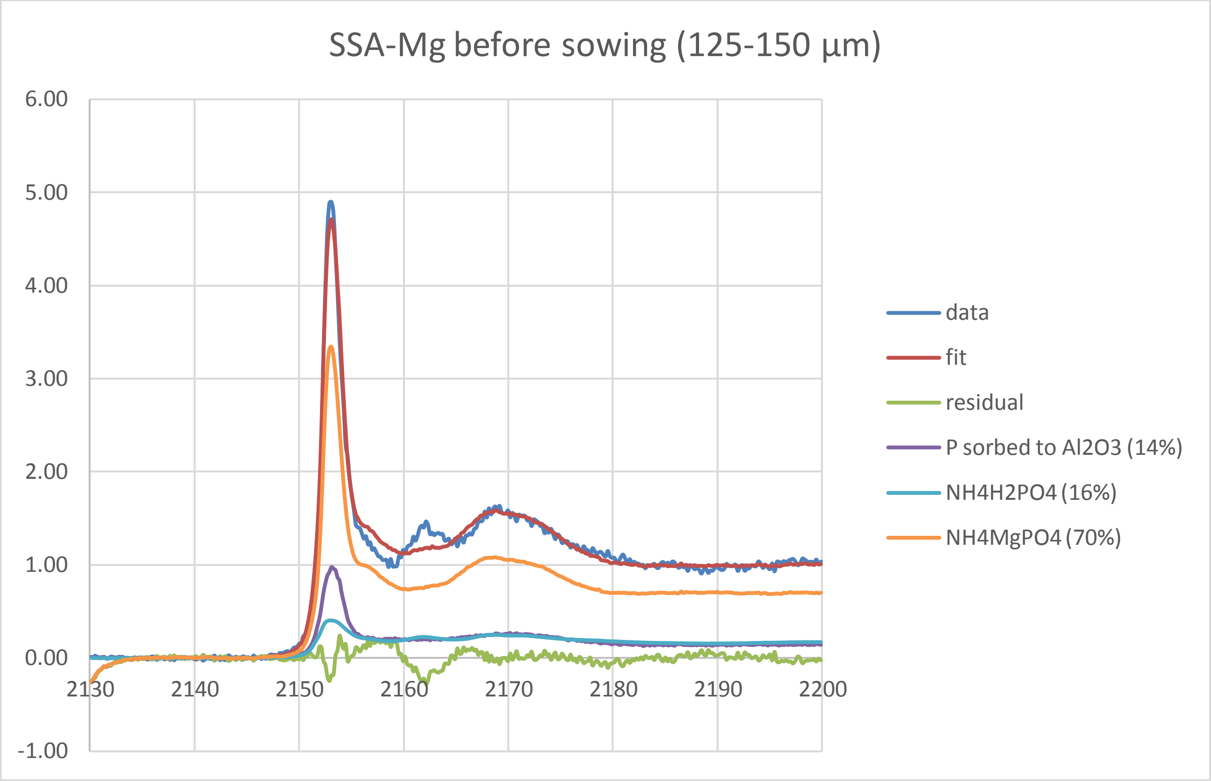

Figure S16:

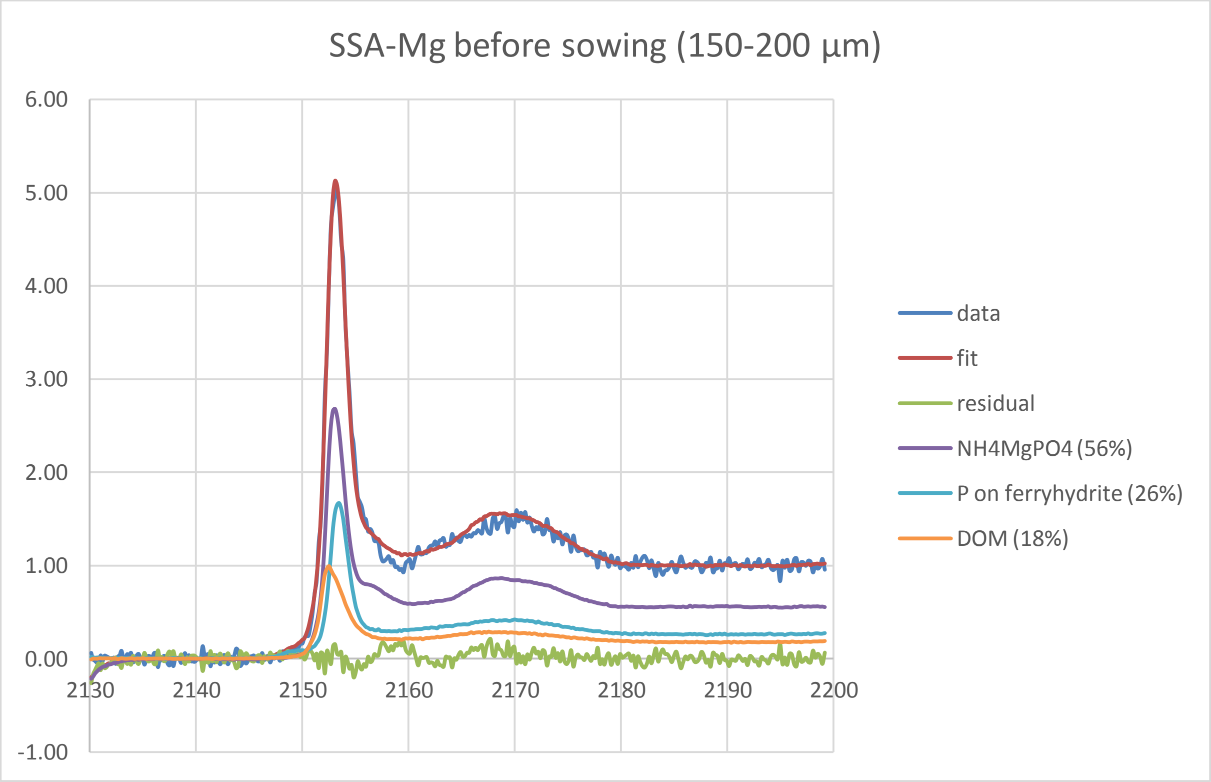

Figure S17:

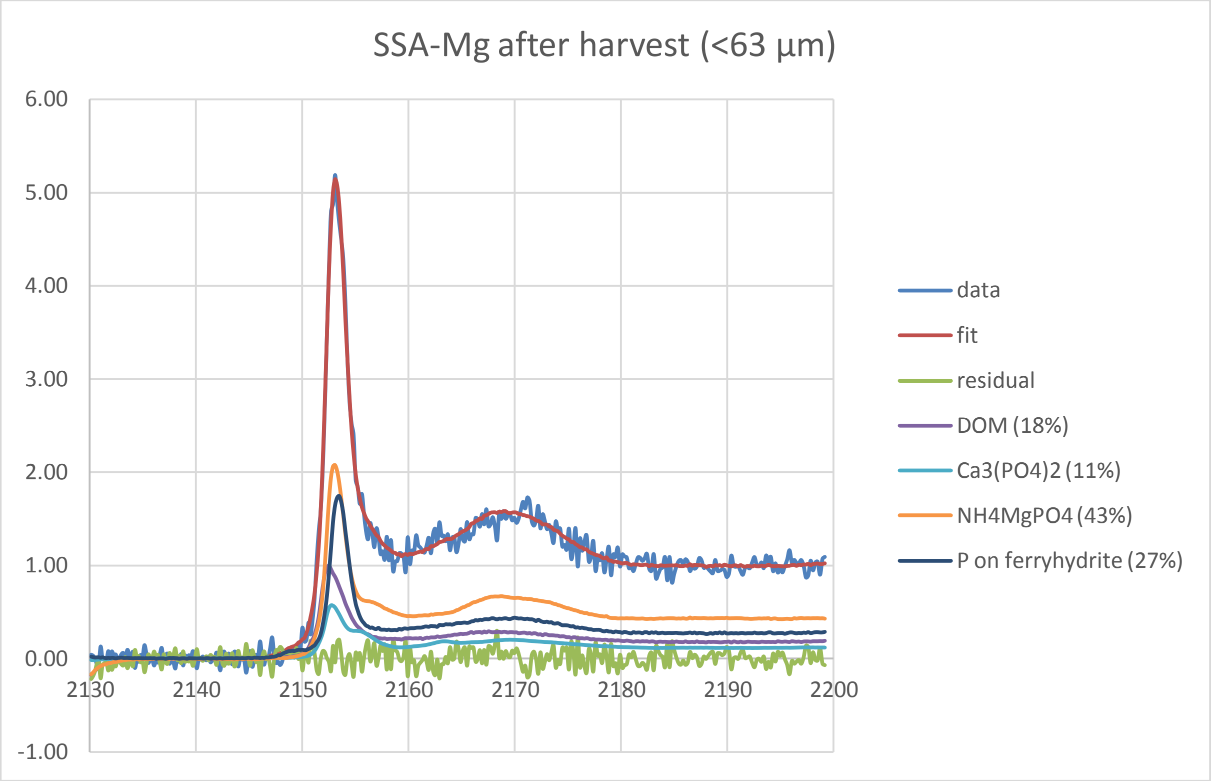

Figure S18:

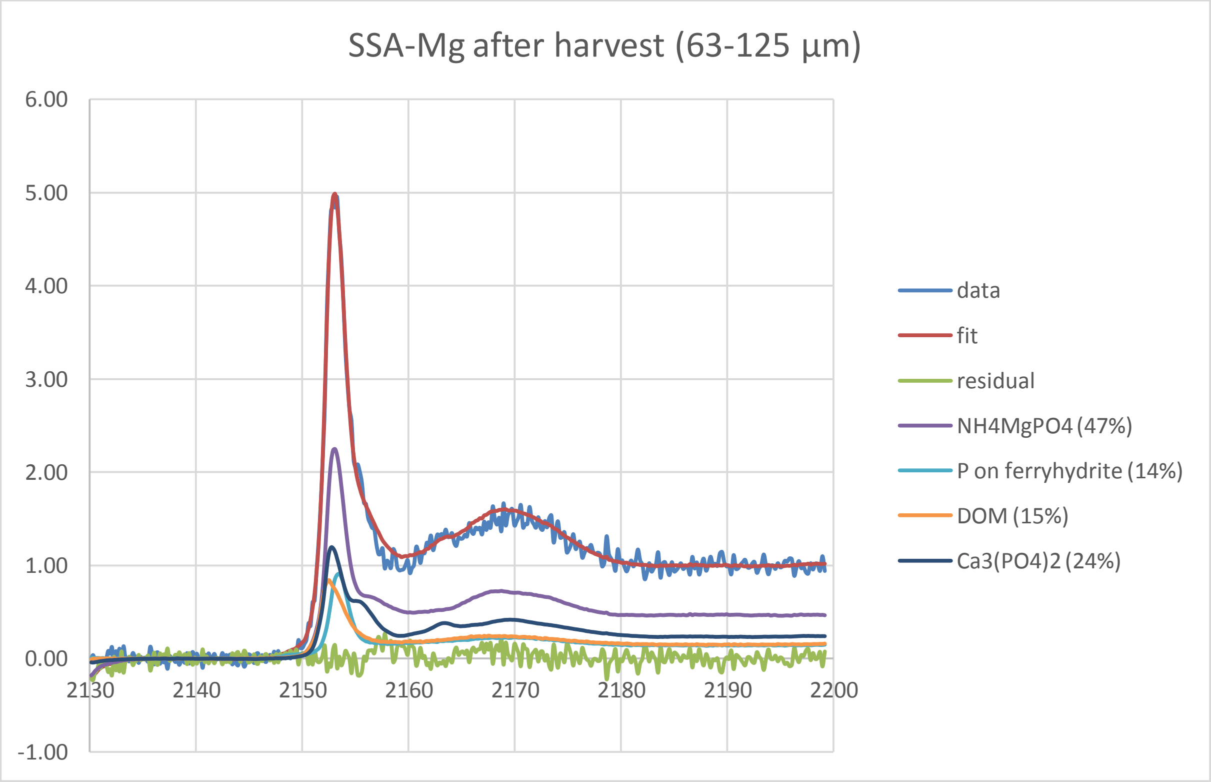

Figure S19:

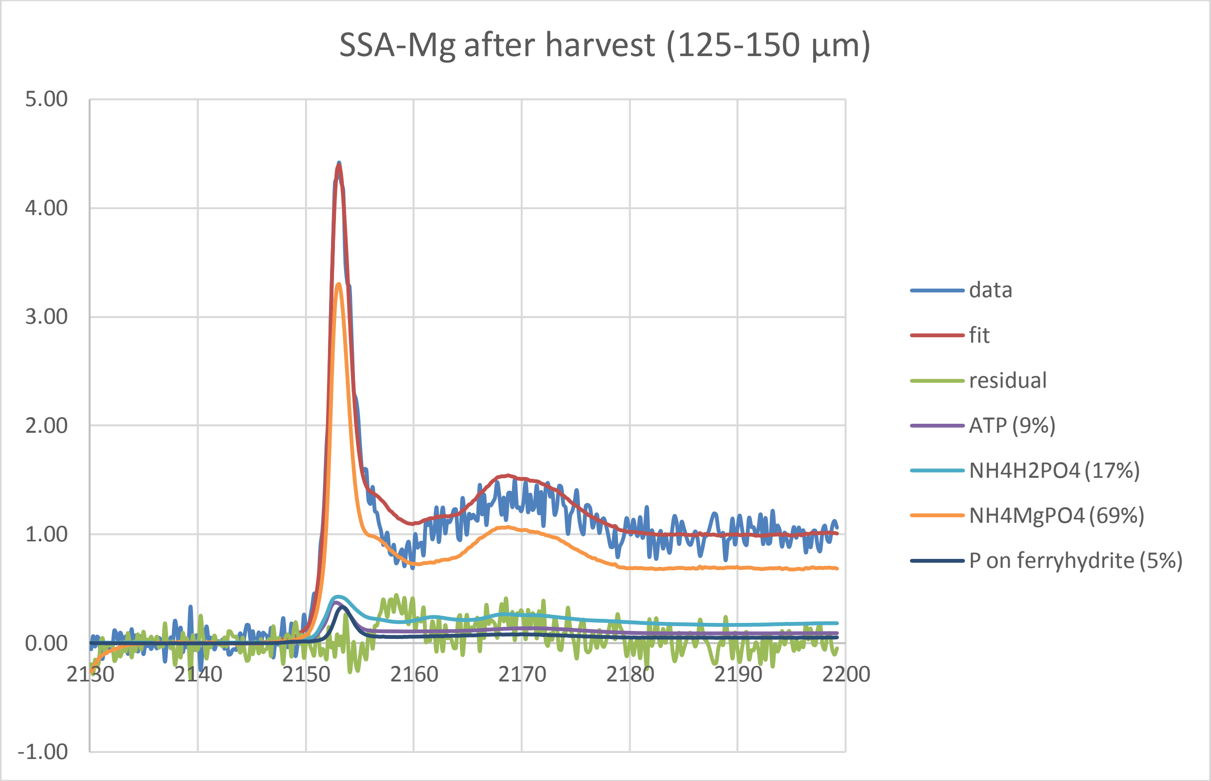

Figure S20:

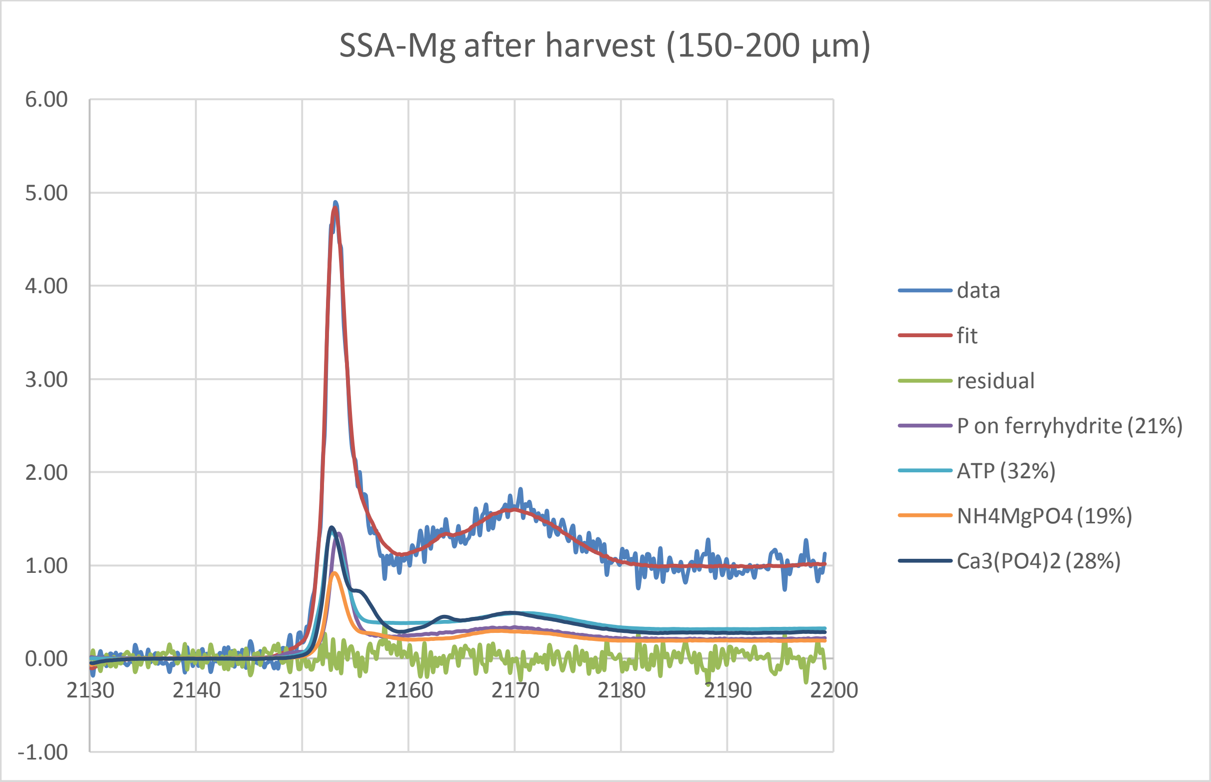

Supplement: Supplementary file 1 — Supplementary material 1 (PDF 2635 kb) [file 13280_2017_973_MOESM1_ESM.pdf]
